# Identification and characterization of a heterotrimeric archaeal DNA polymerase holoenzyme

**DOI:** 10.1038/ncomms15075

**Published:** 2017-05-02

**Authors:** Jiangyu Yan, Thomas R. Beattie, Adriana L. Rojas, Kelly Schermerhorn, Tamzin Gristwood, Jonathan C. Trinidad, Sonja V. Albers, Pietro Roversi, Andrew F. Gardner, Nicola G. A. Abrescia, Stephen D. Bell

**Affiliations:** 1 Department of Molecular and Cellular Biochemistry, Indiana University, Simon Hall MSB, 212 S Hawthorne Dr, Bloomington, Indiana 47405, USA; 2 Sir William Dunn School of Pathology, University of Oxford, South Parks Road, Oxford OX1 3RE, UK; 3 Structural Biology Unit, CIC bioGUNE, CIBERehd, 48160 Derio, Spain; 4 New England Biolabs, 240 County Road, Ipswich, Massachusetts 01938–2723, USA; 5 Department of Chemistry, Indiana University, Bloomington, Indiana 47405, USA; 6 Molecular Biology of Archaea, Institute of Biology II, Microbiology, University of Freiburg, Schaenzlestr. 1, 79104 Freiburg, Germany; 7 Department of Biochemistry, Oxford Glycobiology Institute, University of Oxford, South Parks Road, Oxford OX1 3QU, UK; 8 IKERBASQUE, Basque Foundation for Science, 48013 Bilbao, Spain; 9 Department of Biology, Indiana University, Simon Hall MSB, 212 S Hawthorne Dr, Bloomington, Indiana 47405, USA

## Abstract

Since their initial characterization over 30 years ago, it has been believed that the archaeal B-family DNA polymerases are single-subunit enzymes. This contrasts with the multi-subunit B-family replicative polymerases of eukaryotes. Here we reveal that the highly studied PolB1 from *Sulfolobus solfataricus* exists as a heterotrimeric complex in cell extracts. Two small subunits, PBP1 and PBP2, associate with distinct surfaces of the larger catalytic subunit and influence the enzymatic properties of the DNA polymerase. Thus, multi-subunit replicative DNA polymerase holoenzymes are present in all three domains of life. We reveal the architecture of the assembly by a combination of cross-linking coupled with mass spectrometry, X-ray crystallography and single-particle electron microscopy. The small subunits stabilize the holoenzyme assembly and the acidic tail of one small subunit mitigates the ability of the enzyme to perform strand-displacement synthesis, with important implications for lagging strand DNA synthesis.

## Abstract (web summary)

The current model for B-family DNA polymerases in archaea is one of single-subunit enzymes in contrast to the multi-subunit complexes in eukaryotes. Here the authors show that PolB1 from *Sulfolobus solfataricus* exists as a heterotrimeric complex in cell extracts.

The DNA replication machinery of hyperthermophilic archaea has received considerable interest from academic and biotechnological fields^1^,^2^,^3^. The evolutionary relationship between archaea and eukaryotes has led to the exploitation of the former as model systems for the fundamentally related, yet organizationally more complex, eukaryotic DNA replication machinery. Biotechnological interest lies in the potential exploitation of highly stable DNA polymerases and accessory proteins for DNA amplification procedures^1^. Archaea constitute a diverse range of organisms falling into at least four principal phyla^4^. Much of the replication machinery is conserved across the archaeal domain. For example, the Orc1/Cdc6 initiator proteins, the MCM helicase, processivity clamp PCNA, DNA primase and RFC clamp loader are near ubiquitous^2^,^3^. However, the DNA polymerases of archaea show a surprising level of diversity^5^. DNA polymerases can be classified into one of six families, A, B, C, D, X and Y (refs 6, 7). The X- and Y-family DNA polymerases play roles in DNA repair. The main replicative DNA polymerases of bacteria belong to Family C (refs 8, 9). In contrast, the catalytic subunits of eukaryotic DNA polymerases α, δ and ɛ, responsible for initial RNA primer extension with DNA, lagging and leading strand synthesis, respectively, belong to Family B^10^,^11^,^12^. Notably, all three of these polymerases are multi-subunit complexes. The catalytic subunit typically complexes with a polymerase-specific B-subunit and one or more additional subunits. The additional subunits can facilitate stabilization of the polymerase activity, be targets for regulatory modification and/or mediate interactions with other components of the replisome^13^,^14^.

The nature and phyletic distribution of archaeal DNA polymerases is complex^5^. Most archaea, with the exception of the Crenarchaea, possess a DNA polymerase that belongs to the archaeal-specific family D. Many species additionally encode a family B polymerase, PolB3. Genetic studies in *Thermococcus kodakaraensis* and *Methanococcus maripalaudis* have revealed that the D family polymerase is encoded by essential genes whereas the PolB3 is not essential for viability^15^,^16^. Interestingly, PolB3 is found across the archaeal domain but phylogenetic analyses suggest it has undergone high levels of lateral gene transfer, suggesting it could be derived from an extra-chromosomal element^5^. The Crenarchaea, which lack a family D polymerase, instead possess a signature B-family polymerase, named PolB1, that appears to be the main replicative DNA polymerase. This enzyme was first purified from *Sulfolobus acidocaldarius* (*Sac*) in 1985 (ref. 17). Analysis of the purified enzyme led to the conclusion that it was a single-subunit enzyme with a mass of ∼100 kDa. Subsequent work in *Sulfolobus solfataricus* (*Sso*) led to the cloning of gene for the catalytic subunit, revealing it to be a family B DNA polymerase^18^. The recombinant PolB1 protein possessed activity and has been subsequently investigated by several laboratories in a range of structural and biochemical analyses^19^,^20^,^21^,^22^,^23^,^24^,^25^,^26^,^27^.

In the current work, we re-visit the nature of the native PolB1. Purification of the enzyme from *Sulfolobus* reveals, in contrast to the 30-year old belief, the enzyme is not a single-subunit entity *in vivo*. Rather, it exists as a stable heterotrimer in which the apo-enzyme PolB1 associates with two subunits that we term PolB1-binding protein (PBP) 1 and PBP2. We reconstitute the holoenzyme *in vitro*, confirm that it exists *in vivo*, map the interactions of PBP1 and PBP2 with the catalytic PolB1 subunit, elucidate the structure of the small subunits and the holoenzyme and determine the biochemical consequences of small subunit association on the behaviour of PolB1.

Taken together, our analyses reveal that multi-subunit family B replicative polymerases are not restricted to eukaryotes and thus further underscore the relationship between the *Sulfolobus* replication machinery and that of eukaryotes.

## Results

### Identification of a PolB1-containing holoenzyme

**Figure 1:**
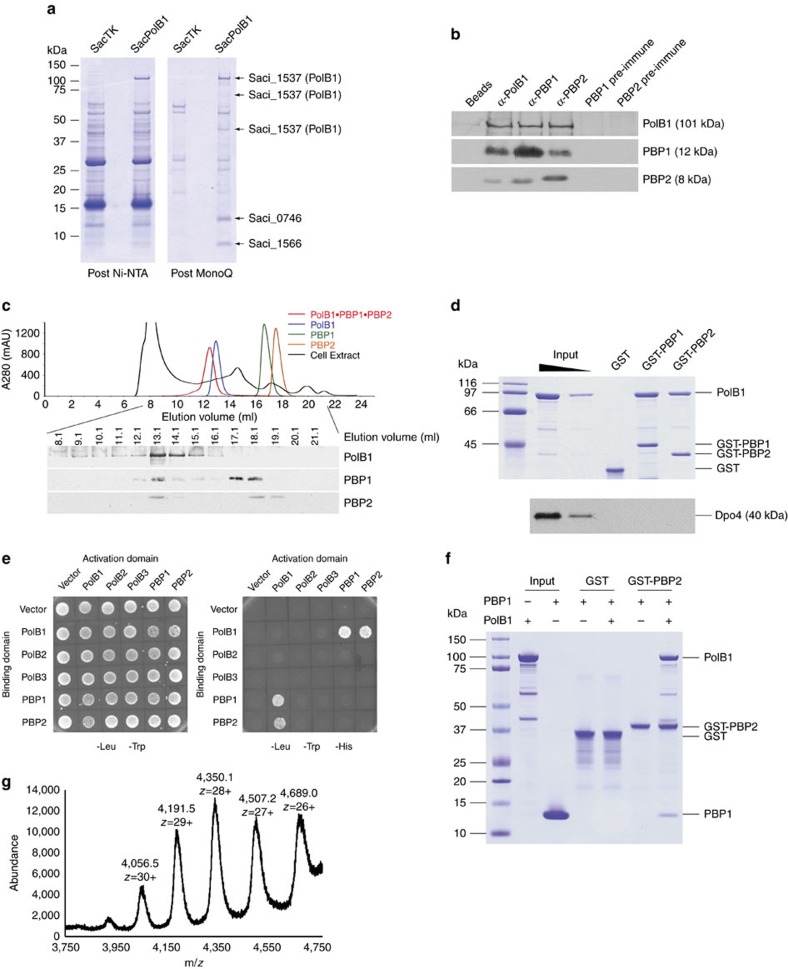
Identification of a PolB1-containing holoenzyme. (**a**) Cell lysates of SacTK (control strain) and SacPolB1 were passed through Ni-NTA and MonoQ columns. The migration of molecular weight markers is indicated on the left of the Coomassie stained 4–12% gradient SDS–PAGE gels. Species identified by mass spectrometry are indicated on the right. (**b**) All three proteins co-immunoprecipitate from *Sso* cell extracts. Anti-sera used in each immunoprecipitation reaction are indicated on the top (‘Beads' denotes no antibody added). Anti-sera used in western blotting are indicated on the right. See Supplementary Fig. 10 for uncropped western blots. (**c**) Gel filtration analysis of cell extracts. Gel filtration column profiles for each recombinant protein (colour coded) and *Sso* cell extract (black) are superimposed. Western blot results of the indicated cell extract gel filtration fractions are presented below the profiles, with the anti-sera used in western blotting indicated on the right. See Supplementary Fig. 10 for uncropped western blots. (**d**) Pull-down of recombinant PolB1 by GST tagged PBP1 and PBP2. Samples were analysed by SDS–PAGE and visualized by Coomassie staining (PolB1) or western blotting using Dpo4 anti-sera. Inputs are 50 and 10%. Molecular weight markers are shown on the left. (**e**) Interaction between PolB1 and PBP1/PBP2 analysed by yeast two hybrid assay. Successful transformation of yeast with fusion constructs is demonstrated by growth on media lacking tryptophan and leucine (left hand panel). Interaction between the indicated fusion proteins is demonstrated by growth on media lacking tryptophan, leucine, and histidine (right hand panel). (**f**) PolB1 mediated pull-down of recombinant PBP1 by GST tagged PBP2. Samples were analysed by SDS–PAGE and visualized by Coomassie staining. Inputs are 50%. Molecular weight markers are shown on the left. (**g**) Native mass determined by electrospray ionization mass spectrometry performed with the PolB1-containing holoenzyme.

To purify endogenous PolB1 we took advantage of the ability to perform epitope tagging of genes on the *Sac* chromosome^28^. We engineered a strain to express a C-terminally myc and hexahistidine-tagged version of PolB1 from the endogenous chromosomal locus. Expression of the tagged protein was confirmed by western blotting with anti-myc and anti-hexaHis tag antibodies. Growth of the strain expressing the tagged PolB1 was indistinguishable from the parental strain and flow cytometry confirmed that the cell cycle was unperturbed (Supplementary Fig. 1). PolB1 was isolated by tandem affinity and ion exchange chromatography. Five polypeptides were uniquely recovered from the tagged strain (Fig. 1a). Mass spectrometry confirmed that the highest molecular weight band of ∼100 kDa was PolB1, as were two smaller species of ∼65 and 40 kDa, presumably arising from limited degradation of the full-length protein. We additionally identified two species, of ∼12 and ∼8 kDa as the products of the Saci_0746 and Saci_1566 open reading frames (hereafter referred to as PBP1 and PBP2, respectively). The small size of the proteins explains why they were not detected in the initial purifications of PolB1 in the 1980s. In those studies the purified polymerase was examined by electrophoresis on relatively low percentage SDS–PAGE gels. Thus, the small subunits would have run off the bottom of the gel^17^,^29^,^30^. Following the cloning of the gene for the catalytic subunit, all subsequent work has utilized the recombinant PolB1 catalytic subunit. Therefore, the majority of studies of *Sulfolobus* PolB1 have been performed with a sub-fraction of a native holoenzyme^19^,^20^,^21^,^22^,^23^,^24^,^25^,^26^,^27^.

The PBP1 and PBP2 proteins are conserved in the *Sulfolobales* and other members of the crenarchaeal phylum (Supplementary Fig. 2) and so, since the majority of our studies of the replisome have been performed with proteins from *Sso*, we chose to study the *Sso* orthologues, the products of the SSO0150 (PBP1) and SSO6202 (PBP2) open reading frames^31^. We purified recombinant forms of these proteins and generated polyclonal anti-sera. Immunoprecipitation reactions using any of the PolB1, PBP1 or PBP2 anti-sera co-precipitated the other two proteins, confirming they form a complex in *Sso* (Fig. 1b). In addition, we performed gel filtration analyses of the individual proteins, the holoenzyme and unfractionated soluble cell-free extract from *Sso*. We monitored elution of the three proteins by western blotting with the appropriate anti-sera and confirmed that all three proteins co-eluted in a peak with a mobility the same as recombinant holoenzyme complex. We note that there are free pools of PBP2 and PBP1 detectable in this analysis (Fig. 1c).

We expressed PBP1 and PBP2 as GST-fusion proteins and tested their ability to interact with recombinant PolB1. As can be seen in Fig. 1d, both GST-PBP1 and GST-PBP2 were capable of direct interactions with PolB1. We also tested their ability to interact with the Y family lesion bypass DNA polymerase, Dpo4 (ref. 32), but failed to detect any specific interactions. Yeast 2 hybrid assays confirmed the PBP1-PolB1 and PBP2-PolB1 interactions but could not detect any direct PBP1–PBP2 interaction (Fig. 1e). We also tested the ability of PBP1 and PBP2 to interact with the *Sulfolobus* family B polymerases using a yeast 2 hybrid assay. *Sulfolobus* species encode three family B polymerase, PolB1, PolB2 (which lacks key residues in its active site and is thus believed to be inactive) and PolB3 (refs 5, 31). We could only detect interaction between PolB1 and either PBP1 or PBP2 (Fig. 1e). The lack of direct PBP1–PBP2 interaction was confirmed by attempts to pull down PBP1 with GST-PBP2. Notably however, when PolB1 was present GST-PBP2 bound to both PolB1 and PBP1, suggesting PBP1 and PBP2 bind to distinct surfaces on PolB1. Thus, PolB1 acts as a bridge between PBP1 and PBP2 (Fig. 1f). Finally, gel filtration analysis with purified PBP1, PBP2 and PolB1 and combinations thereof (Supplementary Fig. 3) confirm the interaction of the three proteins to form a holoenzyme assembly that we will hereafter refer to as PolB1-HE. Native electrospray ionization (ESI) mass spectrometry yields a mass for the complex of 121,660 Da (Fig. 1g), in good agreement with the calculated mass of 121,388 Da for a 1:1:1 stoichiometry of the three subunits in the holoenzyme.

### Structural characterization of PBP1 and PBP2

**Figure 2:**
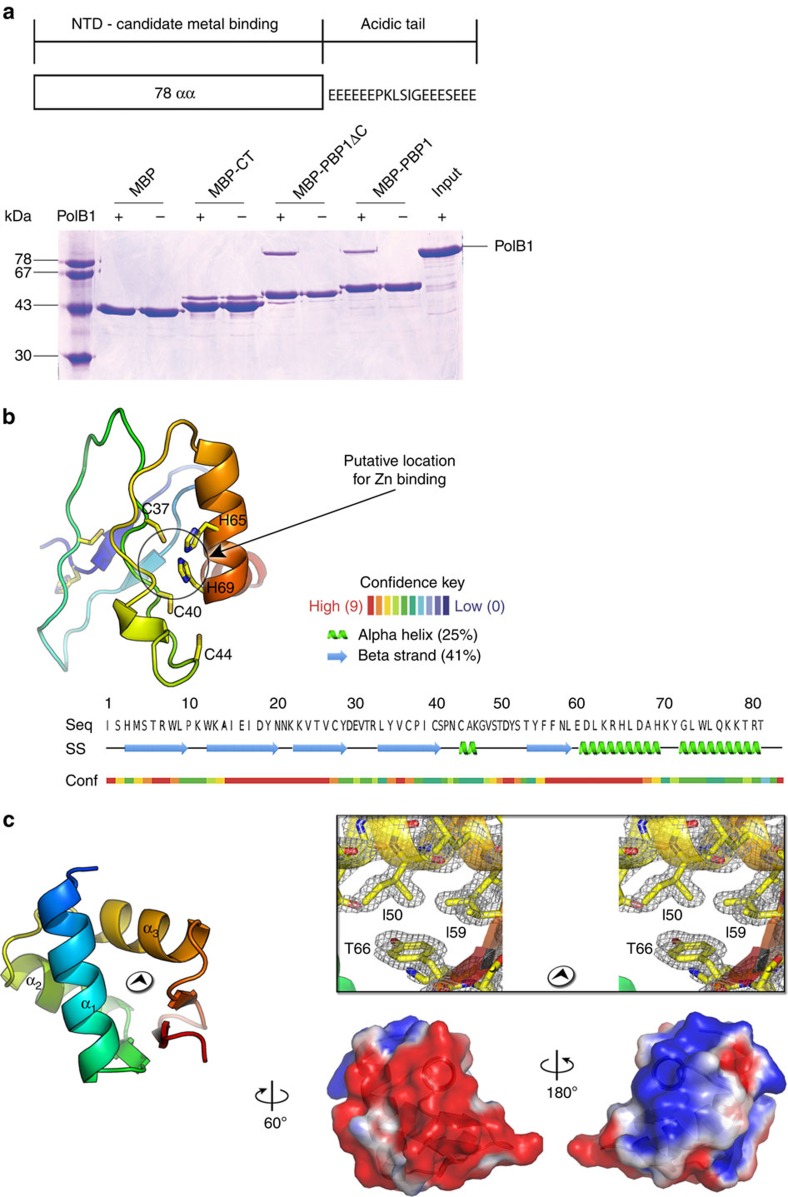
Structural studies of PBP1 and PBP2. (**a**) A diagram of the organization of PBP1 showing the sequence of its acidic tail is shown in the upper panel. The lower panel shows pull-down assays with recombinant PolB1 and MBP-tagged PBP1 and MBP fused to either the first 78 amino acids (MBP-PBP1ΔC) or the C-terminal tail (MBP-CT) of PBP1. Samples were analysed by SDS–PAGE and visualized by Coomassie staining. Molecular weight markers are shown on the left. (**b**) Cartoon representation of PBP1ΔC model obtained from molecular modelling in Phyre2 (ref. 33) and colour coded (in rainbow) from blue (N-terminus) to red (C-terminus), the black circle marks the region where the Zn ion could coordinate with the histidine and cysteine residues; below, the PBP1ΔC sequence with the secondary structure assignment and the confidence of prediction in Phyre. (**c**) Left, PBP2 crystal structure displayed as cartoon colour coded (in rainbow) from blue (N-terminus) to red (C-terminus). Right top, detail in stereoview of the 2Fo-Fc electron density map at 1.35 Å resolution shown as grey mesh contoured at 1.2σ corresponding to the region in the left panel marked with the black circled arrowhead and with residues represented as sticks with overlaid a semi-transparent cartoon representation as left. Right bottom, isopotential surface representation contoured at levels of +1kBT/e (blue) and −1kBT/e (red).

Sequence analysis of PBP1 reveals that the N-terminal 78 residues contain conserved cysteine residues, suggestive of a metal-binding motif. The remaining 19 C-terminal amino acids contain 12 glutamates. We expressed PBP1, PBP1 N-terminal domain and PBP1 C-terminal tail as maltose-binding protein (MBP)-fusion proteins. Using these proteins in affinity chromatography pull-down assays with PolB1 we determined that the N-terminal domain interacted with PolB1, while the C-terminal acidic tail of PBP1 did not stably interact with the polymerase (Fig. 2a). The role of this C-terminal tail will be described below. Attempts to crystallize PBP1 were unsuccessful so we submitted the sequence to the Phyre2 structure prediction server^33^. Phyre2 returned a three-dimensional (3D) molecular model with an *α*+*β* fold with residues 33–69 modelled with 81.5% confidence and containing a conserved C2H2 Zn finger motif (Fig. 2b and Supplementary Fig. 4).

PBP2 was crystallized and the structure determined by multiple wavelength anomalous dispersion (MAD) phasing technique (Table 1 and Supplementary Table 1). The structure, refined to 1.35 Å resolution, reveals a winged helix fold (wH; Fig. 2c, left and right top; Table 1). Interrogation of the protein data bank (PDB) database using the structural comparison DALI server shows that the closest structural homologue to PBP2 is a wH-domain in budding yeast Cdc53 that plays a role in protein–protein interactions. However, wH domains are highly abundant and over 970 significant similarity scores were recovered in the DALI search. The high resolution of the structure visualizes several residues with alternative conformations. Notably C56 shows two side-chain conformers that form a disulfide bridge with the same cysteine of a symmetry related molecule in the lattice. Analysis of PBP2's isopotential surface reveals the molecule has a bipartite charge distribution with the exposed side of helix α1 and the two short antiparallel strands offering a marked positively charged surface, whereas the face of the molecule with the exposed α2 helix presents an almost uniform-negative charge (Fig. 2c, right bottom). We note, however, that we cannot detect any direct interactions between PBP2 and either single- or double-stranded DNA (Supplementary Fig. 5a).

### The architecture of PolB1-HE

**Figure 3:**
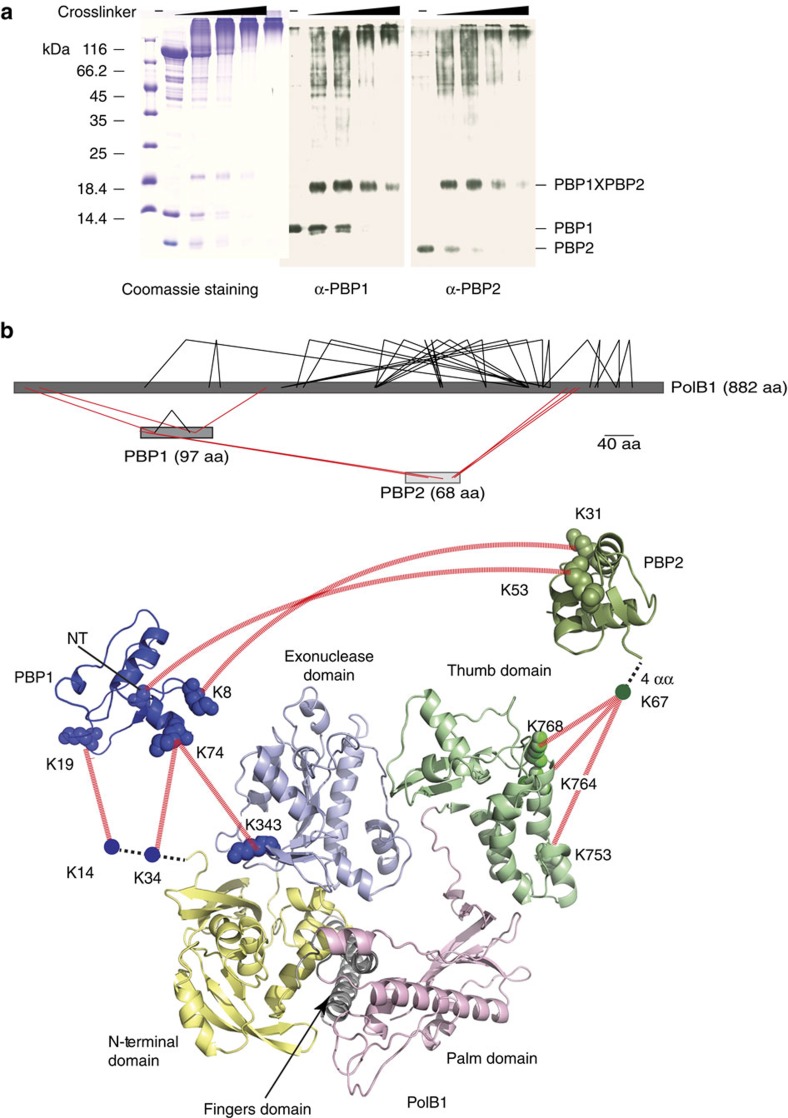
Locations of the sites of PBP1 and PBP2 binding to PolB1. (**a**) Titration of 312 μM, 625 μM, 1.25 mM or 2.5 mM BS^3^ cross-linker with 58 μM holoenzyme complex. Samples were analysed by SDS–PAGE and visualized by coomassie staining (left panel) or western blotting using PBP1 (middle panel) or PBP2 (right panel) anti-sera. Molecular weight markers are shown on the left. (**b**) Cross-linking results of the holoenzyme complex. Upper panel shows an overview of the cross-linked residues within the complex (See also Supplementary Data 3). Intra- and inter-subunit cross-links are indicated in black and red lines, respectively. Inter-subunit cross-links are mapped to the crystal structures of the individual subunits (lower panel). Different domains of PolB1 are colour-coded and labelled. The cross-linked lysine residues are connected with red lines. NT corresponds to the N-terminal amine group. Dashed black lines represent regions of the proteins for which no structural information is available.

To gain insight into the location of the binding sites for PBP1 and PBP2 on PolB1, we performed cross-linking with the homo-bifunctional amine cross-linker bissulfosuccinimidyl suberate (BS^3^). We noted the presence of an abundant species of apparent mass of ∼20 kDa. Western blotting revealed that this band was recognized by anti-sera generated against both PBP1 and PBP2 and the presence of both species within the band was confirmed by mass spectrometry (Fig. 3a, Supplementary Data 1). We searched for cross-linked peptides and identified inter-molecular cross-links between K8 of PBP1 and K31 of PBP2. Although we could not detect direct protein–protein interactions between these proteins in solution (see above), the cross-linking suggests they are within ∼12 Å of one another (the spacer arm on BSH is 11.4 Å) when bound to PolB1. We also observed numerous high molecular weight species. Mass spectrometry revealed the presence of all three polypeptides in these species and we identified several products of intermolecular cross-linking. The positions of the cross-linked amino acids are summarized in Fig. 3b using the structures of PBP1 and PBP2 described above. There is a crystal structure of *Sso* PolB1, however, it has large regions of missing density, particularly in the thumb domain^19^. Accordingly, we generated a homology model of *Sso* PolB1, using the Family B polymerase of *Thermococcus gorgonarius* as the reference^34^. The model is in good agreement with the partial *Sso* PolB1 structure and supplies additional information on the location of missing density in that structure. In summary, PBP2 cross-linked to the thumb domain of the PolB1 while PBP1 was located in the proximity of the exonuclease and N-terminal domains.

**Figure 4:**
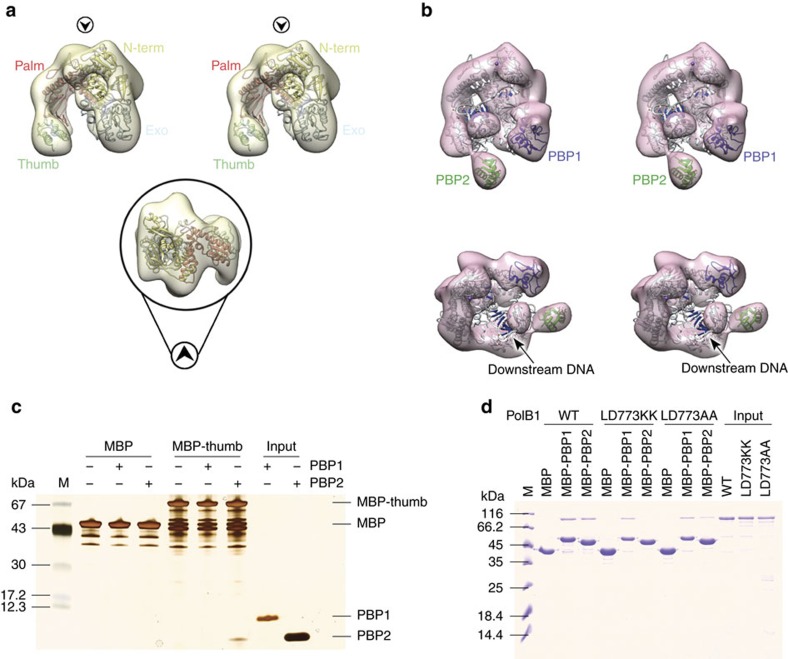
EM and pull downs confirm the binding positions of PBP1 and PBP2. (**a**) Top, stereoview of the EM 3D reconstructed volume of PolB1 (semi-transparent yellow; 22.8 Å resolution, estimated from the Fourier Shell Correlation at 0.5, contoured at 0.04 threshold in Chimera^57^ with the atomic coordinates of *Sso* DNA PolB1 (PDB ID 1S5J) fitted and represented as cartoon (colouring by subdomain is as in Fig. 3b); the map has been post-processed by applying a mask created using the initial reference map (Supplementary Fig. 6b) with soft edges. Black circled arrowheads indicate the relative direction of view of the bottom panel, illustrating the fitting in density of palm and the N-terminal domains. (**b**) Stereoviews of the EM reconstructed density of PolB1-HE holoenzyme (semi-transparent pink; 20.2 Å resolution, as estimated from the FSC at 0.5, contoured at 0.03 threshold) and fitted with the crystal structure of DNA PolB from *Thermococcus gorgonarius* in complex with a DNA primer–template (PDB ID 2VWJ). (**c**) Pull-down of recombinant PBP1 or PBP2 by MBP-tagged PolB1 thumb domain (residues 716–882). Samples were analysed by SDS–PAGE and visualized by silver staining. Molecular weight markers are shown on the left; respective protein bands are indicated on the right. (**d**) Pull-down of recombinant PolB1 wild type or thumb domain mutants by MBP-tagged PBP1 or PBP2. Samples were analysed by SDS–PAGE and visualized by coomassie staining. Molecular weight markers are shown on the left.

Next, we performed negative-stain electron microscopy with apo PolB1 and PolB1-HE that lacked the C-terminal acidic tail of PBP1 and which had been incubated in the presence of a model oligonucleotide primer–template junction. The 3D reconstructed volume of the apo PolB1 structures confirmed the spatial arrangement of the different domains, the N-terminal subdomain, exonuclease subdomain, the palm, finger and the thumb (Fig. 4a and Supplementary Fig. 6). As in the case of the *Sso* PolB1 crystal structure, the volume corresponding to the thumb subdomain only accounted for a fraction of the residues, reiterating the flexibility of this module.

Notably, in the presence of PBP1 and PBP2 we observe additional lobes of density at the thumb domain that, based on our cross-linking and affinity chromatography data, corresponds to PBP2. We propose that the additional lobe in the vicinity of the N-terminal and exonuclease domain corresponds to PBP1 (Fig. 4b). The structure of PolB1 fits well into the EM density, ordering additional residues at the tip of the thumb. PBP2 adds a large wart-like protrusion on the knuckle of the thumb domain (Fig. 4b). We cannot observe significant density corresponding to DNA in the EM structure but this could reflect conformational heterogeneity of the substrate within the holoenzyme. Importantly, the observed position of PBP2 is in agreement with the cross-linking data. We expressed the isolated thumb domain of PolB1 (residues 716–882) as a MBP fusion and confirmed interaction of PBP2 with this domain of PolB1 (Fig. 4c). The EM data indicate that PBP2 associates with the knuckle of the thumb domain of PolB1, accordingly, we aligned PolB1 sequences from a range of Desulfurococcales and Sulfolobales. PolB2 and PolB3 do not interact with PBP2 and we identified residues L772 and D773 that were identical in the PolB1s but divergent in PolB2 and PolB3 (Supplementary Fig. 7, Supplementary Table 4). We speculated, therefore, that these PolB1 signature residues may be of importance for interaction with PBP2. Introduction of point mutations into these conserved residues of the knuckle of the thumb domain reduced or abrogated interaction between PolB1 and PBP2 while not altering the ability of PolB1 to bind PBP1 (Fig. 4d). Furthermore, these mutations minimally impacted on PolB1's DNA polymerase activity (Supplementary Fig. 5b).

### Biochemical characterization of the holoenzyme

**Figure 5:**
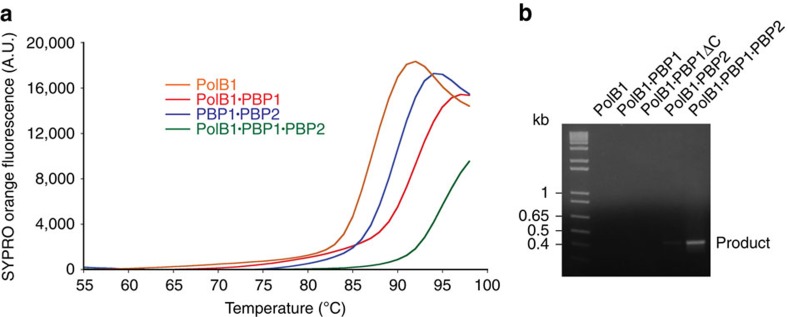
PBP1 and PBP2 enhance the thermostability of PolB1. (**a**) Thermostability of PolB1-HE and sub-assemblies determined by DSF analysis with 40 μM protein in the presence of SYPRO Orange. Fluorescence at emission wavelength 550 nm was recorded in increments of 1 °C. Data are plotted using Kaleidagraph version 4.5. (**b**) PCR activity of PolB1 and the indicated complexes. Reactions contained 100 nM of each enzyme. The ability to generate a PCR product (∼450 bp) was analysed by electrophoresis on a 1% agarose gel. A DNA ladder is on the left with indicated sizes.

We reconstituted the full PolB1-HE holoenzyme and sub-assemblies lacking PBP1 or PBP2 and used differential scanning fluorimetry (DSF) to measure the thermal stability of the various complexes. Addition of either PBP1 or PBP2 increased the thermal stability and formation of the full holoenzyme resulted in further enhancement of the stability of the assembly (Fig. 5a). Indeed, it was impossible to determine the peak of fluorescence in the assay as the polymerase chain reaction (PCR) machine we used had a maximum block temperature of 99 °C. In light of the enhanced thermal stability of the holoenzyme, we next tested the ability of the sub-assemblies to perform PCR. As can be seen in Fig. 5b, the holoenzyme is capable of performing PCR, while the apo PolB1 yields no amplification product.

**Figure 6:**
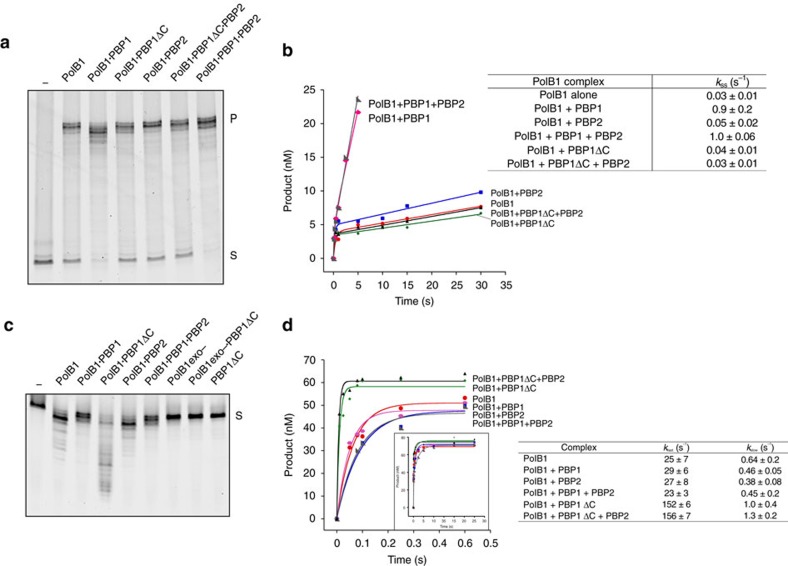
Activity assays for PolB1 assemblies. (**a**) Primer-extension activities of PolB1-HE and sub-assemblies. Reactions contained 2.5 nM enzyme and 50 nM DNA substrate. Reactions were incubated at 75 °C for 5 min. Reaction products were analysed by denaturing PAGE. ‘S' denotes substrate and ‘P' denotes full-length product. (**b**) Steady-state dGTP Incorporation Kinetics for PolB1-HE and sub-assemblies. 5′-FAM-labelled 50 nt primer annealed to template DNA was pre-incubated with polymerase and reacted with dGTP using rapid quench flow (RQF) instrumentation from 0.1–30 s and quenched with 100 mM EDTA. 51 nt dGTP incorporation product was resolved from 50 nt substrate using capillary electrophoresis. 51 nt product was graphed as a function of time to the steady-state burst equation ([Product]=A[1-exp(−*k*_obs_t)+*k*_2_t]) to obtain a *k*_ss_ (*k*_2_/A) for PolB1 (circle), PolB1 + PBP1 (hexagon), PolB1+PBP2 (square), PolB1-HE (right-angled triangle), PolB1+PBP1ΔC (diamond),PolB1+PBP1ΔC+PBP2 (triangle) (left panel). The kinetic parameters for PolB1 sub-assemblies are indicated on the right panel. (**c**) Exonuclease activities of PolB1-HE and sub-assemblies. Reactions contained 1 μM PolB1-HE and subcomplexes with 1 μM DNA substrate. Reaction products were analysed by denaturing PAGE. ‘PBP1ΔC' denotes PBP1 C-terminal tail deletion mutant, ‘PolB1exo-' denotes PolB1 exo-nuclease deficient mutant. (**d**) Pre-Steady-State Exonuclease Kinetics for PolB1 Complexes. 5′-FAM-labelled 50 nt exonuclease oligonucleotide containing a 3′ dGMP was incubated with polymerase using RQF instrumentation from 0.01–30 s and quenched with 1 N H_2_SO_4_+0.25% SDS. 49 nt exonuclease product was resolved from 50 nt substrate using capillary electrophoresis. 49 nt product was graphed as a function of time to a double-exponential equation ([Product]=A_1_[1-exp(−*k*_fast_t)]+A_2_[1-exp(−*k*_slow_t)]) to obtain a *k*_fast_ and *k*_slow_ for dGMP removal by PolB1 (circle), PolB1+PBP1 (hexagon), PolB1+PBP2 (square), PolB1-HE (right-angled triangle), PolB1+PBP1ΔC (diamond), PolB1+PBP1ΔC+PBP2 (triangle). The kinetic parameters for PolB1 sub-assemblies are indicated on the right panel.

Next, we tested the effect of the small subunits on the primer-extension DNA polymerization activity of PolB1. Our initial assays were performed at pH 8.0 and revealed that the presence of the acidic tail of PBP1 exerted an inhibitory effect on the PolB1-HE holoenzyme's ability to polymerize DNA (Supplementary Fig. 5c). This correlated with an impairment of the ability of PolB1-HE to bind a synthetic primer–template junction, as measured by fluorescence polarization assays (Supplementary Fig. 5d). As with the inhibition of polymerization, this inhibition was dependent on the presence of the PBP1 acidic tail. We speculate that the negatively charged tail may be competing with DNA for access to the active site cleft of the DNA polymerase. These observations support our positioning of PBP1 described above. Importantly, the intracellular pH of *Sulfolobus* is weakly acidic and when we performed polymerization studies at pH 6.0 we no longer observed inhibition mediated by PBP1's acidic tail (Supplementary Fig. 5c). Instead, we observed enhanced primer utilization when PBP1's tail was present (Fig. 6a and Supplementary Fig. 5c). The assays were performed under excess substrate conditions and so these data suggest that the tail of PBP1 facilitates recycling of the holoenzyme. The presence of PBP2 led to elevated product yield and enhanced the ability of the holoenzyme to generate full-length product in the presence of PBP1's acidic tail. Next, we performed steady-state kinetic analyses with PolB1, PolB1-HE and the various sub-assemblies (Fig. 6b). These assays used pre-formed enzyme·substrate complex in the presence of an excess of substrate and monitored single-nucleotide incorporation. The initial rate was essentially identical for all complexes, revealing that the rate of phosphodiester bond formation is unaltered by the presence of PBP1 and/or PBP2. However, we saw dramatic differences in rate following this initial burst. The assays monitored single-nucleotide incorporation and so the second phase of synthesis requires disengagement of the enzyme from product and re-engagement with a new substrate molecule. Studies in other systems have revealed that the kinetics of this second phase of incorporation are dominated by the recycling rate of the enzyme^35^,^36^. Notably, the presence of the acidic tail of PBP1 accelerates the rate of post-burst synthesis by 25 to 30-fold. Taken together, these data indicate that the acidic tail of PBP1 facilitates recycling of the holoenzyme. Further evidence for the importance of the acidic tail of PBP1 came from studies of the exonuclease activity of the various complexes (Fig. 6c). In these assays, the enzyme was supplied with a primer–template junction in the absence of dNTPs. Interaction with PBP1 reduced the exonuclease activity compared to the apo PolB1. However, in the absence of the C-terminal tail, PBP1ΔC leads to a substantial stimulation of the exonuclease activity. We confirmed that there was no exonuclease activity inherent to the PBP1ΔC either in isolation or in the presence of an exonuclease-deficient mutant of PolB1. The experiments shown in Fig. 6c employ a substrate in which the primer is fully base paired. Very similar results were obtained with a substrate containing a single-base mismatch at the 3′ end of the primer (Supplementary Fig. 5e). Thus, the small subunits, and particularly the acidic tail of PBP1, impact on both DNA polymerase and exonuclease activities of the enzyme. The qualitative assays were confirmed by measurements of the kinetics of excision of the 3′ nucleotide (dGMP), revealing a ∼6-fold enhancement of dGMP removal effected by inclusion of PBP1ΔC (Fig. 6d). We also determined the influence of the small subunits on the fidelity of the enzyme and found an increase of up to fourfold in fidelity affected by either or both of PBP1 and PBP2 when compared to apo-PolB1 (Supplementary Fig. 8a and b). Notably, the mutations introduced by PolB1 were dominated by deletions, incorporation of either PBP1 and/or PBP2 reduced deletions but elevated the proportion of A/G transitions.

### Strand-displacement synthesis by the holoenzyme

The data presented in Fig. 6 indicate that the acidic tail of PBP1 has a profound effect on the recycling rate of the DNA polymerase; the presence of the tail accelerates this rate by ∼25-fold. We speculated that this might have significance during lagging strand replication, when the polymerase must disengage from a completed Okazaki fragment upon encountering a ‘downstream' fragment and recycle to extend the primer of the next ‘upstream' fragment.

**Figure 7:**
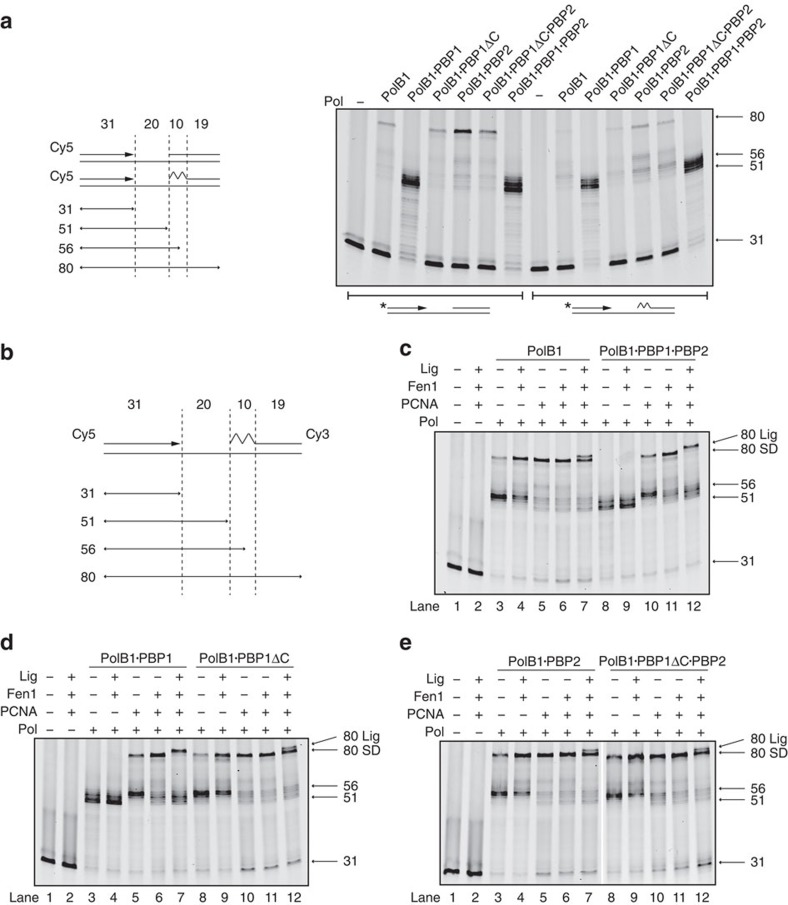
Strand displacement and lagging strand maturation activity of PolB1-HE and sub-assemblies. (**a**) Strand-displacement activity of PolB1-containing holoenzyme and sub-assemblies. Substrate information is illustrated on the left. Primer was labelled with Cy5, jagged region represents the ribonucleotide primer. Reactions contained 10 nM PolB1 sub-assemblies and 50 nM DNA. Reaction products were analysed by denaturing PAGE. Notable product sizes are indicated on the right of the gel. (**b**) Diagram of the substrate used in the following experiments. The primer was labelled with Cy5 and downstream RNA-DNA hybrid oligonucleotide was labelled with Cy3. Lengths of all the oligos and possible reaction products are labelled. The jagged region represents the RNA component of the downstream ‘Okazaki fragment'. (**c**–**e**) Lagging strand maturation assay for all six PolB1 sub-assemblies. Reactions contained 12.5 nM PolB1 sub-assemblies, 200 nM PCNA, 50 nM Fen1, 400 nM Lig1, and 50 nM double-labelled substrate. Reaction products were analysed by denaturing PAGE. The gels were scanned twice with the Cy5 channel (shown in the figure) showing polymerase/strand-displacement products and Cy3 channel showing Fen1-mediated degradation and Lig1 catalyzed ligation products (presented as Supplementary Fig. 8c–e). Product sizes are indicated on the right of the gel. Full strand-displacement product (80 SD) and the ligation product (80 Lig) are also indicated on the right of the gel.

First, we tested the ability of PolB1, PolB1-HE and the sub-assemblies to mediate strand-displacement synthesis on model substrates containing a DNA or RNA·DNA hybrid oligonucleotide as a surrogate ‘downstream' Okazaki fragment. PolB1, PolB1·PBP1ΔC, PolB1·PBP1ΔC·PBP2 and PolB1·PBP2 were all capable of strand-displacement synthesis (Fig. 7a). In marked contrast, however, PolB1·PBP1 and the PolB1-HE holoenzyme, both possessing PBP1's acidic tail, showed enhanced primer utilization but no full-length product. Instead, the majority of products indicated polymerase disengagement at the position of the 5′ end of the downstream fragment or following up to 4–6 nucleotides displacement. Thus, the acidic tail of PBP1 has a considerable impact on the ability of PolB1 to mediate strand-displacement synthesis.

Next, we tested the effect of the sliding clamp PCNA on the polymerase complexes' ability to mediate strand displacement on the RNA·DNA hybrid-containing substrate (Fig. 7b–e, lanes 5 and 10). In all cases, PCNA enhanced the yield of full-length product, even in the presence of PBP1's acidic tail. However, when PBP1's acidic tail was present, the majority of products still corresponded to stalling in the vicinity of the downstream oligonucleotide. PCNA enhanced the ability to the polymerase to extend into the region covered by the RNA component of the downstream oligonucleotide, and, interestingly, the main products coincided with synthesis ending at the position of transition from RNA to DNA in the downstream oligonucleotide.

We then determined whether the limited degree of strand displacement in the presence of PBP1's tail could generate a substrate for cleavage by the Flap Endonuclease, Fen1. In these assays the upstream DNA primer is 5′ labelled with Cy5 and the downstream fragment is 3′ labelled with Cy3, thus we can monitor the fate of both strands. As can be seen in Fig. 7c–e (lanes 6 and 11), Fen1 was able to stimulate the ability of the PolB1-HE and PolB1·PBP1 to mediate strand-displacement synthesis to the end of the template strand in the presence of PCNA. Addition of DNA ligase to the reaction further exposed the role of PBP1's C-terminal tail in governing the strand-displacement synthesis by the polymerase. In all reactions containing polymerase, PCNA, Fen1 and DNA ligase we observe the product of DNA ligation. As this species (labelled ‘80 Lig' in Fig. 7c–e, lanes 7 and 12; Supplementary Fig. 8c–e) contains both fluorophores it is the slowest migrating species on the gels and thus can be resolved from the product of full-length strand-displacement synthesis (labelled ‘80 SD' in Fig. 7c–e). In all reactions lacking the C-terminal tail of PBP1 the ratio of full-length strand-displacement product to ligation product is ∼2:1. However, when PBP1's tail is present, full-length strand-displacement synthesis products are essentially abolished and the only full-length products are the products of ligation. Thus, we propose one of the key roles of PBP1 is to counteract the innate strand-displacement synthesis activity of PolB1 and thereby limit the amount of redundant re-synthesis of DNA during lagging strand maturation.

## Discussion

We have described the identification and characterization of a heterotrimeric archaeal DNA polymerase. Our work, therefore, challenges the long-held belief that archaeal family B DNA polymerases are single-subunit enzymes.

The impacts of the small subunits on the activity of the DNA polymerase are distinct and additive. First, the presence of both subunits causes a large increase in the thermostability of the enzyme compared to the apo-PolB1. In addition, PBP1 modulates the ability of the DNA polymerase to perform strand-displacement synthesis. This role of PBP1 is manifested primarily through its C-terminal acidic tail. The tail acts to govern the strand-displacement activity of PolB1, and thus limits the needless elimination and re-synthesis of DNA in the preceding Okazaki fragment. This may be of particular importance given the small size of archaeal Okazaki fragments^37^. In addition to ensuring the frugality of the enzyme, PBP1 acts to facilitate disengagement of the enzyme as it encounters a downstream Okazaki fragment, and may thus aid in recycling the holoenzyme during lagging strand synthesis.

PBP1 is located in the vicinity of the exonuclease domain of PolB1 and interaction of PBP1ΔC with PolB1 strongly stimulates the enzyme's exonuclease activity. Interestingly, the acidic tail of PBP1 mitigates this stimulation. We note that the effect of PBP1's tail is pH dependent and we speculate that this acidic sequence, tethered close to the PolB1 polymerase and exonuclease active sites, may act as a competitor for DNA binding. In support of this, we observe a pH-dependent reduction in the DNA-binding affinity of the holoenzyme when the acidic tail of PBP1 is present.

Interaction of PBP2 with PolB1 modestly enhances DNA synthesis. In addition, it ameliorates the negative effect of PBP1. The wH domains can play roles in protein–protein and protein–nucleic acid interactions. We have been unable to detect interaction between the isolated protein and either single- or double-stranded DNA. We speculate that PBP2 may facilitate polymerase activity via its interactions with the knuckle on the thumb domain of PolB1 (Fig. 4b), conceivably influencing the position of the tip of the thumb domain and modulating the interaction of this domain of PolB1 with the primer–template junction.

A comprehensive analysis of the phylogenetic distribution of archaeal DNA polymerases by Koonin and colleagues has revealed that PolB1 is found in the Crenarchaea, Thaumarchaea and Korarchaea. Our analysis (Supplementary Fig. 2) reveals that PBP1 and PBP2 are present in the Sulfolobales, Acidilobales and Desulfurococcales and we cannot find an example of an organism that encodes one of the small subunits but not the other. The genes for the two subunits are not closely linked to one another in the genome nor are they linked to that for PolB1. The gene for PBP1 is often found adjacent to one encoding ribokinase and in *Sso* these two genes form a bi-cistronic transcription unit^38^. In contrast, the gene for PBP2 appears to be an isolated ORF within a variable gene environment between species.

In light of our demonstration of the impact of the small subunits on exonuclease and polymerase activities of PolB1 we were initially surprised to be unable to detect genes for PBP1 and PBP2 in Thaumarchaea, Korarchaea nor in crenarchaea of the order Thermoproteales. However, the Thaumarchaea and Korarchaea possess Family D polymerases in addition to PolB1. We also observed that in the Thermoproteales all but one organism encode a PolB1 with a striking N-terminal acidic extension (Supplementary Figs 2 and 9). The sole exception within the Thermoproteales is *Thermofilum pendens* and phylogenetic analyses of PolB1 indicate that, uniquely in this organism, its PolB1 shows a close relationship to the korarchaeal and thaumarchaeal orthologue, suggesting a recent lateral gene transfer. Our data indicate that, in *Sulfolobus*, PBP1 is positioned near the N-terminus of the PolB1 subunit and the acidic tail of PBP1 plays a significant role in modulating polymerase activity. We speculate, therefore, that in the Thermoproteales, this signature acidic N-terminal tail may play a role analogous to PBP1. We note that in eukaryotic DNA pol δ, the p12 subunit possesses a signature acidic domain. Intriguingly, *S. cerevisiae* lacks a p12 orthologue but uniquely possess an acidic embellishment to its B-subunit, providing a precedent for positional malleability of an acidic module within a DNA polymerase holoenzyme^14^.

In conclusion, over 30 years after its first characterization, we reveal that *Sulfolobus* PolB1 is not a single-subunit enzyme as was previously believed, but rather is a heterotrimeric holoenzyme. DNA polymerase holoenzyme assemblies are thus found in all three domains of life. Our work demonstrates how association with accessory subunits can dramatically impact on the core activity of a replicative DNA polymerase in a manner that enhances the efficiency of the resultant assembly during genome duplication.

## Methods

### Protein expression and purification

All proteins were overexpressed in and purified from *Escheria coli* Rosetta cells. *E. coli* cell cultures were grown in LB at 37 °C to OD_600_=0.6 and induced with 1 mM IPTG for 4 h at 37 °C. Cells were collected by centrifugation at 8,983 *g* for 20 min.

Cells expressing PolB1 were lysed in buffer A (10 mM HEPES pH 7.5, 100 mM NaCl, 1 mM dithiothreitol (DTT)) containing Roche Mini-Complete EDTA-free protease inhibitors using French press. The lysate was centrifuged at 34,957 *g* for 30 min and the supernatant was heat-treated at 65 °C for 20 min before further centrifugation (heat-treatment was omitted for PolB1 mutant purification). The supernatant was purified over a HiTrap Heparin column (GE Healthcare), and then further purified over a HiLoad 26/600 Superdex 200 column (GE Healthcare) in buffer A.

Cells expressing PBP1 were lysed in buffer B (20 mM Tris pH 8.8, 300 mM NaCl, 1 mM DTT) using French press. The lysate was centrifuged at 34,957 *g* for 30 min and the supernatant was heat-treated at 70 °C for 25 min before further centrifugation. The supernatant was purified over a HiTrap Q column (GE Healthcare), and further purified over a HiLoad 26/600 Superdex 75 column (GE Healthcare) in buffer B.

Cells expressing PBP2 were lysed in buffer B using French press. The lysate was centrifuged at 34,957 *g* for 30 min and the supernatant was heat-treated at 70 °C for 25 min before further centrifugation. The PBP2 was first enriched using Ni-NTA agarose resin (QIAGEN). The eluted fractions were further purified over a HiLoad 26/600 Superdex 200 column in buffer B.

Cells expressing PBP1ΔC-MBP were lysed in buffer A using French press. The lysate was centrifuged at 34,957 *g* for 30 min and the supernatant was first purified using Amylose Resin (New England Biolabs) in buffer A. The eluted fractions were treated with Factor Xa protease (QIAGEN) at room temperature for 16 h to cleave the MBP tag. The cleavage product was purified over a HiLoad 26/600 Superdex 200 column in buffer A.

For PolB1 complex assembly, the different PolB1 subcomplexes were constituted by mixing each recombinant component and purified over a HiLoad 26/600 Superdex 200 column in buffer A. Alternatively, the holoenzyme complex was built by lysing the cells expressing PolB1, PBP1 and PBP2 together in buffer A using French press. The mixed cell lysate was centrifuged at 34,957 *g* for 30 min and the supernatant was heat-treated at 75 °C for 25 min before further centrifugation. The supernatant was first purified using Ni-NTA agarose resin. The eluted fractions were further purified over a HiLoad 26/600 Superdex 200 column in buffer A.

Cells expressing MBP fusion proteins were lysed in buffer A containing Roche Mini-Complete EDTA-free Protease Inhibitors using French press. The lysates were centrifuged at 34,957 *g* for 30 min and the supernatants were applied to Amylose Resin, which was washed and used for pull-down assays.

Cells expressing GST-fusion proteins were resuspended in buffer B containing protease inhibitors and lysed by French press. The lysates were centrifuged at 34,957 *g* for 30 min. The supernatants were applied to 1 ml glutathione sepharose high performance resin (GE healthcare) pre-equilibrated in buffer B and incubated with agitation for ∼1 h. The resin was washed with buffer B and used for pull-down assays.

PCNA 1-2-3, Fen1 and Lig1 were purified essentially as previously described^21^. Cells expressing PCNA 1-2-3 (three subunits are fused together) were lysed in buffer B containing protease inhibitors using French press. The cell lysate was centrifuged at 34,957 *g* for 30min and the supernatant was heated at 75°C for 25min before further centrifugation. The supernatant was purified by passage through Ni-NTA agarose, and eluted fractions were loaded onto a HiLoad 26/600 Superdex 200 column pre-equilibrated in buffer B.

Cells expressing Fen1 were lysed in (20mM MES pH 6.0, 50mM NaCl, 0.5mM DTT) using French press. The cell lysate was centrifuged and the supernatant was heated at 75°C for 15min before further centrifugation. The supernatant was purified over a HiTrap Heparin column, and then further purified over a HiLoad 26/600 Superdex 200 column.

Cells expressing Lig1 were lysed in buffer B using French press. The cell lysate was centrifuged and the supernatant was heated at 75°C for 25min before further centrifugation. The Lig1 was first enriched using Ni-NTA agarose resin before further purified over a HiLoad 26/600 Superdex 200 column. The protein was then purified with a MonoQ column (GE Healthcare).

### Purification of endogenous *Sac* PolB1

Approximately 3 g of *Sac* biomass from exponentially growing cultures was resuspended in 20 ml 10 mM Tris pH 8, 100 mM NaCl, 10 mM MgCl_2_, 1 mM CaCl_2_, 14 mM β-mercaptoethanol, 0.1% Triton X-100 and complete protease inhibitors, and incubated on ice for 10 min. Cells were lysed by two passages through a French press, before addition of 600 units of DNaseI (Roche) and incubation for a further 30 min on ice. Extracts were centrifuged at 34,957 *g* for 30 min to remove cell debris. The buffer composition of the supernatant was adjusted to 10 mM Tris pH 8, 300 mM NaCl, 10 mM imidazole, 14 mM β-mercaptoethanol before application to 2 ml of Ni-NTA resin, equilibrated in the same buffer. Bound protein was washed with 20 mM imidazole before elution with 500 mM imidazole. PolB1-containing fractions were diluted sixfold in 10 mM Tris pH 8, 1 mM DTT, applied to a 1 ml MonoQ column and eluted over a 15 ml linear gradient of 50–1,000 mM NaCl. PolB1-containing fractions were concentrated by TCA precipitation and analysed by SDS-PAGE. Indicated gel bands were excised with a scalpel, digested with trypsin and analysed by liquid chromatography–mass spectrometry (LC–MS/MS; Sir William Dunn School of Pathology Central Proteomics Facility). The highest-scoring protein identified by MASCOT is indicated for each band.

### Immunoprecipitation and western blotting

*Sso* cells were lysed in buffer C (20 mM Tris pH 7.5, 150 mM NaCl, 5 mM MgCl_2_, 10% Glycerol 1 mM DTT) using French press. The lysate was centrifuged at 34,957 *g* for 30 min and the supernatant was passed through a 0.45 μm filter (Sartorius Stedim Biotech). Immunoprecipitation was carried out by adding 10 μl of anti-PolB1, PBP1 or PBP2 sera to 100 μg of cell extracts to make 100 μl reactions in buffer C. The mixtures were incubated on a rotating wheel at room temperature for 1 h before the addition of 10 μl nProtein A Sepharose (GE Healthcare). The reactions were further incubated at room temperature for 1 h. The beads were washed four times by bead pelleting and resuspension in 500 μl buffer C, followed by boiling in 1 × SDS–PAGE loading buffer. For western blotting, the proteins were transferred from SDS–PAGE gels to nitrocellulose membranes (PerkinElmer), which were then probed with 1:1,000 dilutions of anti-PolB1, PBP1 or PBP2 serum.

### Whole-cell extract gel filtration assays

*Sso* whole-cell extracts were prepared as described above in buffer D (50 mM HEPES pH 7.5, 150 mM KCl, 0.2 mM EDTA, 5 mM MgCl_2_, 10% Glycerol, 1 mM DTT, 0.05% NP-40) containing Roche Mini-Complete EDTA-free protease inhibitors. Cells were lysed in buffer D using French press and the lysate was incubated on ice for 10 min with the addition of 10 μl Benzonase (Novagen) before centrifugation at 34,957 *g* for 10 min. The soluble cell extract was further clarified by passing the supernatant through a 0.45 μm filter. For gel filtration, the extract was treated with 24 μg ml^−1^ ethidium bromide (to prevent protein–DNA interactions) and 500 μl of the treated extract was applied to a Superdex 200 HR 10/30 column in buffer E (10 mM Tris pH 8.0, 150 mM NaCl). The recombinant proteins, PolB1-HE, PolB1, PBP1 and PBP2, were also applied to the column, separately. The fractions from the cell extracts gel filtration were tested by western blot with 1:1,000 dilutions of anti-PolB1, PBP1 or PBP2 serum.

### Analytical gel filtration

Samples (100 μl) containing 20 mM Tris pH 8, 300 mM NaCl and the indicated proteins were incubated for 10 min at room temperature, before loading onto a Superdex 200 HR 10/30 column equilibrated in 20 mM Tris pH 8, 300 mM NaCl. Fractions of 1 ml were collected and analysed by SDS-PAGE on a 4–12% gradient gel (Invitrogen). Molecular weight calibration was performed using a low molecular calibration kit (GE healthcare).

### GST pull-down assays

Reactions were assembled in 1.5 ml Eppendorf tubes by mixing ∼10 μg of GST-fusion protein and 10 μg of target in 100 μl TBST (10 mM Tris pH 8, 150 mM NaCl, 0.1% Tween 20). The reactions were incubated at room temperature for 30 min. TBSTM was used when testing GST-PCNA fusion proteins (TBST supplemented with 5 mM MgCl_2_). Beads were recovered by centrifugation and washed three times with 500 μl TBST/TBSTM, followed by boiling in 1 × SDS–PAGE loading buffer. Proteins were resolved on a 10 or 12% polyacrylamide gel and visualized with Coomassie staining or western blotting with the indicated anti-sera.

### MBP pull-down assays

Reactions were assembled in 1.5 ml Eppendorf tubes by mixing Amylose Resin beads (bound with MBP fusion proteins) and target protein in buffer A to a total volume of 100 μl each. The reactions were incubated in a Thermomixer (Eppendorf) with shaking at room temperature for 30 min. Beads were then washed four times by bead pelleting and resuspension in 1 ml buffer A, followed by boiling in 1 × SDS–PAGE loading buffer.

### Yeast 2 hybrid assay

*Saccharomyces cerevisiae* haploid strain AH109 was transformed according to the Matchmaker protocol (Clontech) with plasmids encoding the indicated proteins fused to the GAL4 activation domain or GAL4 DNA-binding domain. Transformants were selected by growth on SC-Leu-Trp plates after 3 days at 30 °C. To test for interactions, each diploid strain was replica plated onto both SC-Trp-Leu and SC-Trp-Leu-His agar; this was achieved either by direct restreaking of a single colony, or by plating 5 μl of a single colony grown in YPED media to an OD_600_ of 0.1. Growth is shown after 3 days at 30 °C; growth on media lacking histidine after this time is considered confirmation of a protein–protein interaction.

### Enzyme polymerase chain reaction

Fifty-microlitre reactions containing 120 mM Tris pH 8.8, 2 mM MgSO_4_, 10 mM KCl, 10 mM (NH_4_)_2_SO_4_, 0.1% Triton X -100, 100 μg ml^−1^ nuclease-free BSA, 200 μM dNTPs, 400 nM Primers, 250 ng Template DNA and 100 nM enzyme complexes were assembled. The thermal cycle was programed as follow: 95 °C for 20 s; 30 cycles of 95 °C for 10 s, 55 °C for 20 s, 72 °C for 90 s; 72 °C for 60 s. The products were examined by electrophoresis on a 1% (w/v) agarose gel.

### Fluorescence anisotropy

Hundred-microlitre reactions containing 10 mM Tris pH 8 or pH 6, 100 mM NaCl, 10 nM 6-FAM-labelled primer–template junction and a range of concentrations of PolB1-HE and sub-assemblies were assembled (in triplicates) in 96-well plates. The reactions were incubated at 50 °C for 10 min before analysis in a Synergy 2 plate reader at the same temperature. Data were plotted using Kaleidagraph version 4.5 (Synergy Software) and fit to single–site-binding model. For PBP2, the procedure is essentially the same except the buffer is 1 × TBS.

### Kinetic assays

Steady-state dGTP incorporation and pre-steady-state exonuclease kinetics were performed for PolB1-HE and sub-assemblies as follows:^39^. For steady-state dGTP incorporation kinetics, primer/template sequences are as follows: Primer 5′-FAM-AGT GAA TTC GAG CTC GGT ACC CGG GGA TCC TCT AGA GTC GAC CTG CAG G*T-3′ (where * denotes location of phosphorothioate bond), Template 5′-AAG TAT GAA AGT AGG ACA CCT GCA GGT CGA CTC TAG AGG ATC CCC GGG TAC CGA GCT CGA ATT CAC T-3′, and final reaction conditions 40 nM primer/template, 10 nM PolB1 and 100 μM dGTP in reaction buffer (50 mM Tris-HCl pH 6.8, 10 mM MgCl_2_, 10 mM DTT). For exonuclease kinetics, single-stranded DNA oligonucleotide was as follows: 5′-FAM-AGT GAA TTC GAG CTC GGT ACC CGG GGA TCC TCT AGA GTC GAC CTG CAG *TG-3′ where * denotes location of phosphorothioate bond, and final reaction conditions were 100 nM ssDNA and 400 nM PolB1 in reaction buffer. In addition to the PolB1 RQF time courses, steady-state and pre-steady-state RQF time courses were carried out for each of the 5 PolB1-containing complexes. Steady-state time courses were fit the steady-state burst^39^. Pre-steady-state exonuclease kinetic time courses were fit to a double-exponential equation to obtain rate of dGMP removal^40^.

### Activity assay substrate preparation

Fluorescence-labelled oligonucleotides and templates were synthesized by IDT (Integrated DNA Technologies) and are listed in Supplementary Table 3. Primer–template junction substrates were assembled by annealing 1 μM-labelled primer to two-fold molar excess template in NEB buffer 4. Strand-displacement substrates were assembled by annealing 1 μM-labelled primer to 2-fold molar excess of template and 4-fold molar excess of downstream oligonucleotides. Lagging strand maturation substrates were assembled by annealing 1 μM-labelled upstream primer to 1.25-fold molar excess of template and 1.5-fold molar excess of labelled downstream oligonucleotide. Annealing process was carried out as previously described by heating the oligos to 99°C for 10min before gradual cooling to 4°C^21^.

### DNA polymerization assays

Twenty-microlitre reactions containing 50 mM Tris pH 6.8 (for pH comparison, Tris pH 6 and 8 were used), 40 mM KCl, 10 mM DTT, 100 μg ml^−1^ BSA, 10 mM MgCl_2_, 100 μM dNTPs, 50 nM Cy5-labelled substrate, and indicated amounts of varies PolB1-HE and sub-assemblies were assembled. Reactions were incubated at 60 °C for 5 min (unless otherwise indicated) before quenched by the addition of 20 μl loading buffer (8 M Urea, 1 × TBE), followed by adding of 0.5 μl 100 μM template complementary DNA and boiling at 100 °C for 5 min. The products were analysed by electrophoresis through a 12% polyacrylamide, 8 M Urea, 1 × TBE gel and visualized using Typhoon scanner (GE Healthcare).

### Exonuclease assays

Exonuclease reactions were assembled essentially the same as polymerization assays but containing 50 nM enzymes and no dNTPs. Reactions were incubated at 60 °C for 10 min before quenched by the addition of 20 μl Loading buffer (8 M Urea, 1 × TBE), followed by adding of 0.5 μl 100 μM template complementary DNA and boiling at 100 °C for 5 min. The products were analysed by electrophoresis through a 12% polyacrylamide, 8 M Urea, 1 × TBE gel and visualized using Typhoon scanner.

### Strand-displacement assays

Twenty-microlitre reactions containing 50 mM Tris pH 6.8, 10 mM DTT, 100 μg ml^−1^ BSA, 10 mM MgCl_2_, 100 μM dNTPs, 50 nM Cy5-labelled substrate, 100 nM PCNA (if presented) and 10 nM varies PolB1-HE sub-assemblies. Reactions were incubated at 60 °C for 5 min before quenched by the addition of 20 μl loading buffer (8 M urea, 1 × TBE), followed by adding of 0.5 μl 100 μM template complementary DNA and boiling at 100 °C for 5 min. The products were analysed by electrophoresis through a 12% polyacrylamide, 8 M Urea, 1 × TBE gel and visualized using Typhoon scanner.

### Lagging strand maturation assays

Twenty-microlitre reactions containing 50 mM Tris pH 6.8, 40 mM KCl, 10 mM DTT, 100 μg ml^−1^ BSA, 10 mM MgCl_2_, 100 μM dNTPs, 1 mM ATP, 50 nM double-labelled substrate, 200 nM PCNA, 50 nM Fen1, 400 nM Lig1, and 12.5 nM of PolB1-HE and sub-assemblies. Reactions were incubated at 60 °C for 10 min before being quenched by the addition of 20 μl loading buffer (8 M Urea, 1 × TBE), followed by addition of 0.5 μl 100 μM template complementary DNA and boiling at 100 °C for 5 min. The products were analysed by electrophoresis through a 12% polyacrylamide, 8 M Urea, 1 × TBE gel and visualized using Typhoon scanner.

### Polymerase fidelity assay

Gapped plasmid pSJ3 was prepared essentially as follows^41^. Twenty-microlitre reactions containing 50 mM Tris pH 6.8, 40 mM KCl, 10 mM DTT, 100 μg ml^−1^ BSA, 10 mM MgCl_2_, 100 μM dNTPs, 1 mM ATP, 200 nM PCNA, 50 nM Fen1, 400 nM Lig1, 2 nM Gapped pSJ3 and 12.5 nM of polymerase under investigating. Reactions were incubated at 60 °C for 10 min, after that, 9 μl of the reaction was directly quenched by adding 0.4 μl of 10% SDS and the another 9 μl of the reaction was treated with 20 U EcoRI (New England Biolabs) at 37 °C for 30 min before quenching with 0.4 μl of 10% SDS. The samples were then analysed by 0.8% agarose gel electrophoresis. The last 2 μl of the reaction were transformed to *E. coli* 10β cells, which were then plated on LB plates (containing 40 μg ml^−1^ X-gal, 250 μM IPTG and 100 μg ml^−1^ Ampicillin) followed by blue/white screening. Background mutations were determined by either carrying out the same protocol above without adding polymerases to the reactions or directly transforming gapped pSJ3 to *E. coli* cells. Plasmids were recovered from all white colonies and subjected to DNA sequencing.

### Crystallization of PBP2 and X-ray data collection

Crystallization nanodrops were set-up using a Mosquito robot with vapour diffusion hanging drop technique at a 1:1 and 3:1 ratio protein:mother liquor, and incubated at 21 °C. PBP2 at OD_280_=42.7 mg ml^−1^ in buffer containing 20 mM HEPES pH 7.5, 0.3 M NaCl, 1 mM MgCl_2_ and 1 mM beta-merpatoethanol was mixed with the commercial solution of 20% w/v PEG 3350 and 0.2 M NaNO_3_ (Pact-premier, Molecular Dimensions). Crystals appeared after 4 days and grew for 3 days. Crystals were soaked for about 2 min in a solution of the mother liquor (20% PEG 3350 and 0.2 M NaNO_3_) containing 20 mM GdCl_3_, cryo-protected with 25% glycerol and flash frozen in liquid nitrogen for consequent data collection. The soaked crystal was scanned across the LIII Gd edge and three data sets (set-1, -2, -3 in Table 1 and Supplementary Tables 1 and 2) were collected at the peak (*λ*=1.71076 Å), inflexion point (*λ*=1.71145 Å) and high-energy remote (*λ*=1.70371 Å) wavelengths at the ID29 beamline at European Synchrotron Radiation Facility (ESRF; France) diffracting to 2.2 Å resolution. A further native crystal obtained by mixing PBP2 at OD_280_=17.7 mg ml^−1^ in 20 mM HEPES pH 7.5, 0.3 M NaCl, 1 mM MgCl_2_ and 1 mM beta-merpatoethanol with 0.1 M Potassium thiocyanate and 30% w/v Polyethylene glycol monomethyl ether 2000 (Hampton Index) was flash frozen as described (no GdCl_3_ present) and collected at the I02 beamline at Diamond (UK) and diffracted to 1.35 Å resolution (Table 1).

### X-ray data processing and structure determination

The MAD data for PBP2 protein were indexed and scaled in *P*3_1_21 (or *P*3_2_21) *a*=*b*=62.1 Å *c*=35.7 Å with the xia2 autoprocessing suite^42^,^43^ (Table 1, Supplementary Table 1). The anomalous difference Patterson map calculated for the peak data at 2.7 Å showed two major peaks in the Harker section *w*=1/3 the presence of two peaks. The programme phenix.hyss^44^ was used to find two Gd sites against the pk1 data set anomalous differences; the positions and B factors of the two sites were then refined against all three MAD data sets (Supplementary Table 2) in SHARP^45^ to final parameters: Gd1 *x*=0.6574 y=0.0981 *z*=0.1086 occ=0.18 B=53.6Å2; Gd2 *x*=0.6010 *y*=0.1020 *z*=0.1365 occ=0.21 B=80.75 Å^2^. Supplementary Table 2 contains also the SHARP phasing statistics. Solvent flattening with 50% solvent content was performed with the programme Solomon^46^ in *P*3_1_21 (CC=35%) and with the enantiomorphic constellation of heavy atoms in *P*3_2_21 (CC=58%). The latter phases were used for autobuilding in ArpWarp^47^, giving a model with 57 out 77 residues and *R*=24% *R*_free_=28.6% in REFMAC5 (ref. 48). Manual building in Coot^49^ and refinement in autoBUSTER^50^ against the 2.2 Å remote-1 data set gave a model that, at later stage, was used for phasing the 1.35 Å resolution native data. This native structure was then refined in Phenix^51^ (Table 1).

### Holoenzyme sample preparation and EM data collection

For the negative-stain visualization of the naked PolB1 (∼100 kDa) a glow-discharged carbon-coated grid (CF-300_Cu; EMS, USA) was placed onto 10 μl droplet of 10 μg ml^−1^ PolB1 and allowed to sit for 1 min, washed with MilliQ water and then stained using 2% uranyl formate solution. Excess liquid was removed by gently touching the grid-side with Whatmann filter paper. A similar procedure was used for the preparation of negative-stain grids of the holo-complexes PolB1:PBP1-ΔC:PBP2:DNA (∼130 kDa) at 10 μg ml^−1^ (Supplementary Fig. 5a). Complex with DNA was prepared by incubating the complex with double-stranded DNA at room temperature for 1 h. The mixture was then applied to GE S200 16/60 gel filtration column, the fraction containing protein–DNA complex was used for grid preparation.

A total of 108 images for apo-PolB1 and 400 images for the holoenzyme·DNA PolB1-HE were collected. All data were acquired with a JEOL JEM-2200FS electron microscope operated at 200 kV at a magnification of 90,201 and with underfocus between 1.3–1.7 μm. Images were recorded with an ULTRASCAN 4000, 4 K × 4 K CCD camera (Gatan Inc.) and resulting sampling of 1.66 Å per pixel at the specimen.

### EM processing and 3D reconstruction

Two-dimensional (2D) negative-stain images of the apo-PolB1 and PolB1-HE were pre-processed and phase flipped in XMIPP^52^ before particle extraction and 2D and 3D classification routines in Relion software^53^. About 20,000 and 110,000 particles were extracted, respectively, for the apo-PolB1 and the PolB1-HE using a box of 128 × 128 pixels (Supplementary Fig. 6a). Also the Scipion framework software^54^ was used for image processing and different strategies at the classification step were tested in each case.

For the apo-PolB1 after initial 2D classification and manual inspection of useful classes and class members, 3D classification (18,020 particles) in five distinct classes using a circular mask of 170 Å diameter was performed in C1 symmetry. As starting reference volume for the projection matching routine in Relion, the electron density map corresponding to the crystal structure of PolB1 from *Sso* (PDB ID 1S5J) filtered to 50 Å resolution was used (Supplementary Fig. 6b). One out of the five 3D classes showed a large representation with 15,213 particles which then were analysed. Further particles were removed based on manual inspection and on the values of the rlnLogLikeliContribution. The 14,582 particles were then used for the auto-3d refinement routine in Relion. The angular step was set 3.75° and the refinement converged after 10 cycles (Supplementary Fig. 6c, left). The gold-standard Fourier shell correlation (FSC) calculated at iteration 10 (Supplementary Fig. 6c, right) indicates a resolution of 22.8 Å at 0.5 FSC criteria (14.6 Å at 0.143 FSC criteria).

In the case of the PolB1-HE sample we expected a much higher degree of heterogeneity, and therefore after initial manual polishing of particles we straightly set-up a 3D classification routine in ten classes. Also in this case we used as a reference volume the map corresponding to the naked PolB1 filtered to 50 Å resolution (Supplementary Fig. 6b). As expected several of the classes were spurious and each 3D class required careful examination and cross-comparison at the graphics. Out of the ten 3D classes, one class displayed a clean and meaningful volume in terms of interpretability in light of the PolB1 crystal structure alone and at the same time with marked differences. These differences consisted into two clear additional globular densities in proximity of the thumb domain and in an enlarged region close to the exonuclease domain. These densities were weaker than the main core density implying a certain degree of mobility. The angular assignments of the 11,758 particles composing this class were further refined in Relion (Supplementary Fig. 6d, left) producing the final volume shown in Fig. 4b. The resolution as estimated by the gold-standard FSC at the converged iteration is, respectively, 20.2 Å at 0.5 FSC and 14.9 Å at 0.143 FSC criteria (Supplementary Fig. 6d, right).

### Fitting of atomic models into EM maps

The atomic models of PBP1-ΔC and PBP2 were analysed in COOT^49^ and the isopotential surface of the latter calculated with the PDB2PQR and APBS software^55^,^56^ and displayed using Pymol (The PyMol Molecular Graphics System, Schrodinger, LLC). The three-dimensional volumes for the apo-PolB1 and PolB1-HE were analysed in Chimera^57^ and contextualized with the crystal structures of PolB1 of *Sso*^19^ (PDB ID 1S5J) and of *Thermococcus gorgonarius* in complex with a DNA primer–template^58^ (Tgo-Pol; PDB ID 2VWJ) together with the models of PBP1-ΔC and PBP2 investigated in this study. The above atomic models were fitted as a rigid-body in Chimera. As the X-ray structure of Tgo-Pol was complexed with uracil-containing DNA, the holo PolB1-HE density was also inspected in presence of modelled DNA (Fig. 4b).

### Native mass spectrometry

The PolB1-HE complex was prepared in 50 mM ammonium acetate at a concentration of 4.2 μM. The sample was infused into a Synapt G2S mass spectrometer equipped with a nano ESI source at 0.5 μl per min. The source conditions were as follows: capillary voltage=2.67 kV; source temperature=100 °C; sampling cone=40 V; source offset=80 V; desolvation temperature=150 °C; nanoflow gas pressure=0.6 Bar. To promote declustering of solvent ions from the complex, the trap and transfer collision energies were maintained at 8 V. The data was processed in MassLynx (Waters Corporation, MA, USA). Results from one minute of scanning was averaged and smoothed using the Savitzky Golay algorithm with four channels and 20 rounds of smoothing^59^. To determine the mass of the intact complex, the data was processed using MaxEnt 1 (Waters Corporation, MA, USA), with a resolution of 10 Da per channel and damage model with a uniform Gaussian half height of 40 Da.

### Cross-linking followed by mass spectrometry

Hundred-microlitre reaction containing 57 μM Holoenzyme complex and 0.625 mM BS^3^ cross-linker (Thermo Scientific) in buffer F (10 mM HEPES pH 7.5, 150 mM NaCl) was incubated at room temperature for 30 min before quenched by addition of Tris-HCl pH 7.5 to 10 mM, followed by further incubation at room temperature for 15 min. The reaction was then mixed with 400 μl 8 M Guanidine Hydrochloride (in 10 mM Tris-HCl pH 8.0), followed by incubation with 40 μl Ni-NTA agarose resin (pre-washed with buffer F) on a thermomixer at room temperature for 30 min. The resin was then washed twice with buffer F and the cross-linked product was eluted with 40 μl 500 mM imidazole (in buffer F). The elution was applied to a SDS–PAGE gel and the desired bands were excised for Mass Spectrometry. Individual candidate bands were excised from silver stained gels, diced into 1 mm squares and washed with 25 mM ammonium bicarbonate. Cysteine residues were reduced by incubating for 45 min at 57 °C with 2.1 mM dithiothreitol. These reduced side chains were then alkylated with 4.2 mM iodoacetamide for 1 h in the dark at 21 °C. A solution containing 12 ng μl^−1^ trypsin, in 25 mM ammonium bicarbonate was added to cover the gel pieces and the samples were digested for 12 h at 37 °C. The resulting peptides were extracted with two aliquots of 50% acetonitrile, 5% formic acid. The samples were dried down and analysed using an LTQ Velos mass spectrometer (Thermo Fisher Scientific, Waltham MA) coupled to an Eksigent HPLC operating in ‘top eight' data-dependent MS/MS selection. The peptides were separated using a 75 micron, 15 cm column packed in-house with C18 resin (Michrom Bioresources, Auburn, CA, USA) at a flow rate of 300 nl per min. A 1 h gradient was run from buffer A (2% acetonitrile, 0.1% formic acid) to 60% buffer B (100% acetonitrile, 0.1% formic acid). Samples were also analysed on an LTQ Orbitrap XL (Thermo Fisher Scientific, Waltham MA) coupled to an Eksigent HPLC operating in ‘top five' data-dependent MS/MS selection. Spectra were analysed using Protein Prospector version 5.18.22 (prospector.ucsf.edu). Variable modifications allowed were: protein N-terminal acetylation; N-terminal Gln cyclization; oxidation of Met. Carbamidomethylation of cysteine residues was set as a fixed modification and one missed tryptic cleavage was allowed. BS^3^ was allowed to form cross-links between lysine side chains as well as the protein amino terminus. A hydrolyzed version of bis(sulfosuccinimidyl)suberate was allowed to form ‘dead end' cross-links with these residues. For data acquired in the LTQ Velos, the mass tolerances were set to 0.6 Da for the precursor and product ions. For data acquired in the LTQ Orbitrap XL, the mass tolerances were set to 10 p.p.m. and 0.6 Da for the precursor and product ions, respectively. The data was searched against the sequences for PolB1, PBP1, and PBP2. An expectation value cutoff of 0.05 was used and all results were manually inspected.

### Thermal stability assay

The assay is based on the differential scanning fluorimetry (DSF) method^60^. Twenty-five-microlitre reactions containing 10mM HEPES pH 7.5, 100mM NaCl, SYPRO Orange (Life Technologies) and 4 mM protein to be tested were assembled in a 96-well PCR plate. The reactions were heated from 30°C to 99°C in a Real-Time PCR system (Eppendorf Mastercycler Realplex). Fluorescence was recorded in increments of 1°C at 550nm emission wavelength. The data points were plotted using Kaleidagraph version 4.5.

### Data availability

Protein Data Bank: Atomic coordinates and structure factors have been deposited under the accession number 5N35 (Gd-PBP2) and 5N41 (PBP2).

Electron Microscopy Bank: Maps corresponding to the Apo and holo structures have been deposited with the accession numbers EMD-3458 (apo-PolB1), EMD-3462 (PolB1-HE).

## Additional information

**How to cite this article:** Yan, J. *et al*. Identification and characterization of a heterotrimeric archaeal DNA polymerase holoenzyme. *Nat. Commun.* **8,** 15075 doi: 10.1038/ncomms15075 (2017).

**Publisher's note:** Springer Nature remains neutral with regard to jurisdictional claims in published maps and institutional affiliations.

## Supplementary Material

Supplementary Information Supplementary figures, supplementary tables and supplementary references.

Supplementary Data 1 Crosslinks detected by mass spectrometry. Gel band "lower" is the ~25 kDa species containing PBP1 and PBP2 and "upper" is the crosslinked holoenzyme.

## Figures and Tables

**Table 1:** Data collection and refinement statistics for PBP2 (SSO6202).

	**Native**	**Gadolinium derivative**		
*Data collection*				
Space group	*P*3_2_21	*P*3_2_21		
Cell dimensions				
*a*, *b*, *c* (Å)	61.8, 61.8, 35.4	62.1, 62.1, 35.7		
*α*, *β*, *γ* cell (°)	90, 90, 120	90, 90, 120		
		Peak-1	Inflection-1	Remote-1
Wavelength (Å)	0.97949	1.71076	1.71145	1.70371
Resolution (Å)	30.9–1.35	31.1–2.7	31.1–2.7	31.1–2.2
*R*_merge_	0.046 (0.339)	0.07 (0.30)	0.07 (0.29)	0.06 (0.88)
*CC_1/2_	(0.839)			
Mean *I*/*σ*(*I*)	28.2 (3.1)	33.7 (8.1)	35.2 (8.0)	31.6 (1.3)
Completeness (%)	95.2 (68.6)	100 (100)	99.9 (99.0)	99.0 (90.7)
Redundancy	4.8 (3.0)	18.5 (17)	18.4 (16.7)	17.0 (8.7)
				
*Refinement*				
Resolution (Å)	30.8–1.35			31.1–2.2
*N*^o^ reflections	16,503			3,740
*R*_work_/*R*_free_	0.160 (0.214)/0.198 (0.250)			0.227 (0.377)/0.275 (0.396)
*N*^o^ atoms				
Protein	516			473
Ligand/ion	1			48
Water	75			31
*B*-factors				
Protein	27.1			34.0
Ligand/ion	17.9			68.0
Water	51.4			36.7
R.m.s.d.'s				
Bond lengths (Å)	0.009			0.010
Bond angles (°)	0.954			1.290
Ramachandran plot				
Favoured (%)	98.3			98.5
Allowed (%)	1.7			1.5
Outliers (%)	0			0

*Highest resolution shell is shown in parenthesis.

^*^
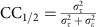
 (ref. 61).
