# Supplementary material for: Identification and characterization of a heterotrimeric archaeal DNA polymerase holoenzyme
Source: Nat Commun. 2017 May 2;8:15075. doi: 10.1038/ncomms15075 (PMC5418573; doi:10.1038/ncomms15075)
Supplement: Supplementary Information — Supplementary figures, supplementary tables and supplementary references. [file ncomms15075-s1.pdf]

Supplementary Table 1. MAD data collection and phasing statistics of the SSO6202 $GdCl_3$ soaked crystal.

MAD data collection statistics

Beamline	ID29@ESRF								
Dataset	peak-1	peak-2	peak-3	infl-1	infl-2	infl-3	remote-1	remote-2	remote-3
Wavelength (Å)	1.71076			1.71145			1.70371		
Resolution (Å)	31.1-2.7	31.1-2.6	31.1-2.3	31.1-2.7	31.1-2.7	31.1-2.3	31.1-2.2	31.1-2.7	31.1-2.3
Observations	44041	37250	37250	43098	35161	51973	67201	36144	53150
Unique observations	2385 (227)	2550 (170)	3478 (265)	3648 (219)	2191 (184)	3463 (260)	3963 (336)	2310 (189)	3524 (272)
Rmerge	0.07 (0.30)	0.05 (0.29)	0.05 (0.17)	0.07 (0.29)	0.05 (0.15)	0.04 (0.13)	0.06 (0.88)	0.05 (0.15)	0.04 (0.11)
Rmeas	0.07 (0.32)	0.05 (0.36)	0.05 (0.21)	0.07 (0.31)	0.05 (0.17)	0.05 (0.17)	0.07 (0.98)	0.07 (0.17)	0.04 (0.14)
‹I/σ(I)›	33.7 (8.1)	37.4 (3.5)	37.2 (7.3)	35.2 (8.0)	44.5 (11.1)	42.6 (9.7)	31.6 (1.3)	45.1 (10.7)	44.7 (12.0)
Completeness %	100 (100)	94.1 (69.1)	95.6 (75.7)	99.9 (99.0)	98.1 (86.4)	95.4 (74.6)	99.0 (90.7)	97.9 (87.0)	96.0 (77.1)
Multiplicity	18.5 (17)	14.6 (4.6)	15.0 (4.7)	18.4 (16.7)	16.0 (7.5)	15.0 (4.9)	17.0 (8.7)	15.6 (6.8)	15.1 (5.4)
Anomalous multiplicity	10.1 (9.1)	7.9 (2.5)	8.1 (2.6)	10.1 (8.8)	8.7 (3.9)	8.1 (2.7)	9.1 (4.6)	8.5 (3.7)	8.1 (2.9)
Anomalous completeness	100 (100)	92.7 (62.0)	94.2 (69.5)	99.8 (98.5)	97.8 (85.5)	94.1 (67.8)	98.6 (87.6)	97.2 (82.0)	94.8 (73.6)
Dano CC	0.82 (0.08)	0.86 (0.11)	0.88 (-0.02)	0.61 (0.03)	0.68 (-0.07)	0.73 (-0.14)	0.42 (-0.22)	0.53 (0.08)	0.61 (-0.05)

Supplementary Table 2. SHARP phasing statistics for resolution 31.1- 2.2 Å (in parenthesis values up to 6.6 Å)

Dataset	Phasing Power (isomorphous)		Phasing Power (anomalous)	FOM	
	Acentrics	Centrics	Acentrics	Acentrics	Centrics
Peak-1	-	-	2.87 (5.15)	-	-
Peak-2	-	-	2.92 (6.22)	-	-
Peak-3	-	-	2.93 (4.90)	-	-
Inflection-1	0.52 (1.81)	0.63 (1.32)	1.44 (3.19)	-	-
Inflection-2	1.15 (2.79)	1.46 (2.36)	1.82 (3.49)	-	-
Inflection-3	0.65 (2.12)	0.79 (1.66)	1.71 (3.13)	-	-
Remote-1	0.37 (1.58)	0.52 (1.35)	1.31 (4.82)	-	-
Remote-2	1.23 (3.81)	1.61 (2.64)	1.98 (5.28)	-	-
Remote-3	0.51 (1.71)	0.65 (1.48)	2.07 (5.11)	-	-
Overall				0.300 (0.765)	0.498 (0.939)

Supplementary Table 3. Oligonucleotides used in this study.

Oligo Name	Sequence (5' to 3')
PolB1_For	CATGACCCATGGCTAAGCAACTTACCTTATTTG
PolB1_Rev	CATGACCTCGAGTTAACTATTTCCTTTACTTGGGTATG
PolB1_notag mutation	CCCAAGTAAAGGAAATAGTTAACTCGAGCACCACCACCACC ACCACTGAGAT
PBP1_For	CATGACCATATGTCAACGAGATGGCTACC
PBP1_Rev	CATGACCTCGAGCGTAAATCCTCCTACTATTAATATCTTAC
PBP2_For	CATGACCATATGTCTGTTAATCAGAAGGAAATTG
PBP2_Rev	CATGACCTCGAGCTTCTTGTCAGTAGATTTCCTCAC
PolB1_LD/KK	ACTAAAAAACAAAGGATACAATAAGAAGGAATTAGCGTTTA AAGTAATGC
PolB1_LD/AA	ACTAAAAAACAAAGGATACAATGCGGCCGAATTAGCGTTTA AAGTAATGC
PolB1_Thumb_For	GATACCATGGGCATGTTAGTGAAGAAGAGAAAC
PolB1_Thumb_Rev	GCACGTCGACTTAACTATTTCCTTTACTTGGGTA
Template_long	TTAAAGTTAGGTGGCGGACTCTGCCTCAAATCGTGTAATGAT GCCATGCGACCTCTGACAACGTGACCGCAGCCCACCTG
Template mismatch	TTAAAGTTAGGTGGCGGACTCTGCCTCAAATCGTGTAATGAT GCCATGCAACCTCTGACAACGTGACCGCAGCCCACCTG
Template_short	TTAAAGTTAGGTGGGGGACTCTGCCTCAAGACCTCTGACAA CGTGACCGCAGCCCACCTG
Cy5 primer	Cy5/CAGGTGGGCTGCGGTCACGTTGTCAGAGGTC
Top_DNA	TTGAGGCAGAGTCCGCCACCTAACTTTAA
Top_hybrid	rUrUrGrArGrGrCrArGrAGTCCGCCACCTAACTTTAA
Top_hybrid Cy3	rUrUrGrArGrGrCrArGrAGTCCGCCACCTAACTTTAA/Cy3
Templ_complementary	CAGGTGGGCTGCGGTCACGTTGTCAGAGGTCTTGAGGCAGA GTCCGCCA
EM oligo1	ACAGGTAAGCAGTCCGCG
EM oligo2	GCGGACTGCTTACDDC
FA_label	[6FAM]CAGGTGGGCTGCGGTCACGTTGTCAGAGGTC
FA_template	GTGTAATGATGCCATGCGACCTCTGACAACGTGACCGCAGC CCACCTG

Supplementary Table 4. Accession numbers for PolB1 sequences in Supplementary Figure 7a. SSOPolB2 and SSOPolB3 are included.

Protein	Species	Accession Number
SSOPolB3	*Sulfolobus solfataricus*	WP_009988892.1
SSOPolB2	*Sulfolobus solfataricus*	WP_009989138.1
ASA	*Acidilobus saccharovorans*	WP_013266050.1
FFO	*Fervidicoccus fontis*	AFH42863.1
IGIS	*Ignicoccus islandicus*	WP_075050453.1
IGHO	*Ignicoccus hospitalis*	WP_011998724.1
THCE	*Thermogladius cellulolyticus*	WP_014737097.1
SMAR	*Staphylothermus marinus*	WP_011839163.1
SHEL	*Staphylothermus hellenicus*	WP_013143881.1
THAG	*Thermosphaera aggregans*	WP_013130178.1
DEMU	*Desulfurococcus mucosus*	WP_013562821.1
DEFE	*Desulfurococcus fermentans*	WP_014768161.1
DEKA	*Desulfurococcus kamchatkensis*	WP_052290773.1
PYFU	*Pyrolobus fumarii*	WP_014027250.1
MEYE	*Metallosphaera yellowstonensis*	WP_009075591.1
MECU	*Metallosphaera cuprina*	WP_013736984.1
MESE	*Metallosphaera sedula*	WP_048060164.1
ACHO	*Acidianus hospitalis*	WP_048054919.1
SSOPolB1	*Sulfolobus solfataricus*	WP_009991059.1
SIS	*Sulfolobus islandicus*	WP_012711569.1
STO	*Sulfolobus tokodaii*	WP_010979471.1
SAC	*Sulfolobus acidocaldarius*	WP_011278353.1

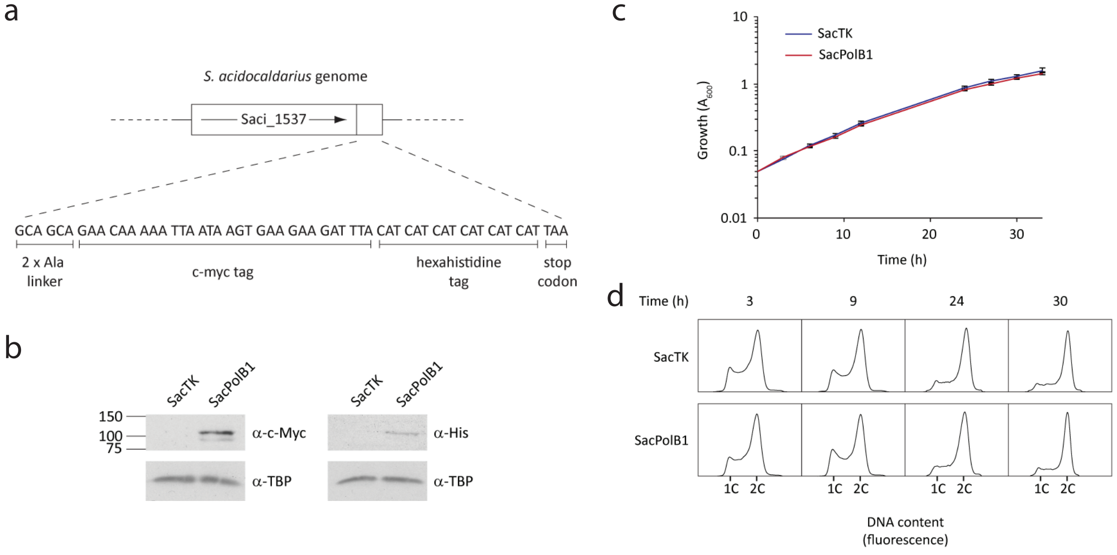

Supplementary Figure 1. Characterization of a *S. acidocaldarius* strain in which the gene for PolB1 (*saci_1537*) is modified with a dual affinity tag.

(a) Schematic of chromosomal modification of the gene for S. acidocaldarius PolB1 (Saci_1537). The endogenous gene was replaced by a copy containing the additional 3' sequences illustrated in the expanded view.

(b) Western blot analysis using anti-c-Myc and anti-his-tag antibodies of parental (SacTK) and modified (SacPolB1) strains to confirm the expression of the dual-tagged protein. TBP was used as a loading control.

(c) Comparison of growth of parental (SacTK) and modified (SacPolB1) strains over time, measured by absorbance at 600 nm. The scale on the y-axis is logarithmic.

(d) FACS analysis of the two strains above at the indicated time points. 1C and 2C indicate the 1 and 2 chromosome content cell populations.

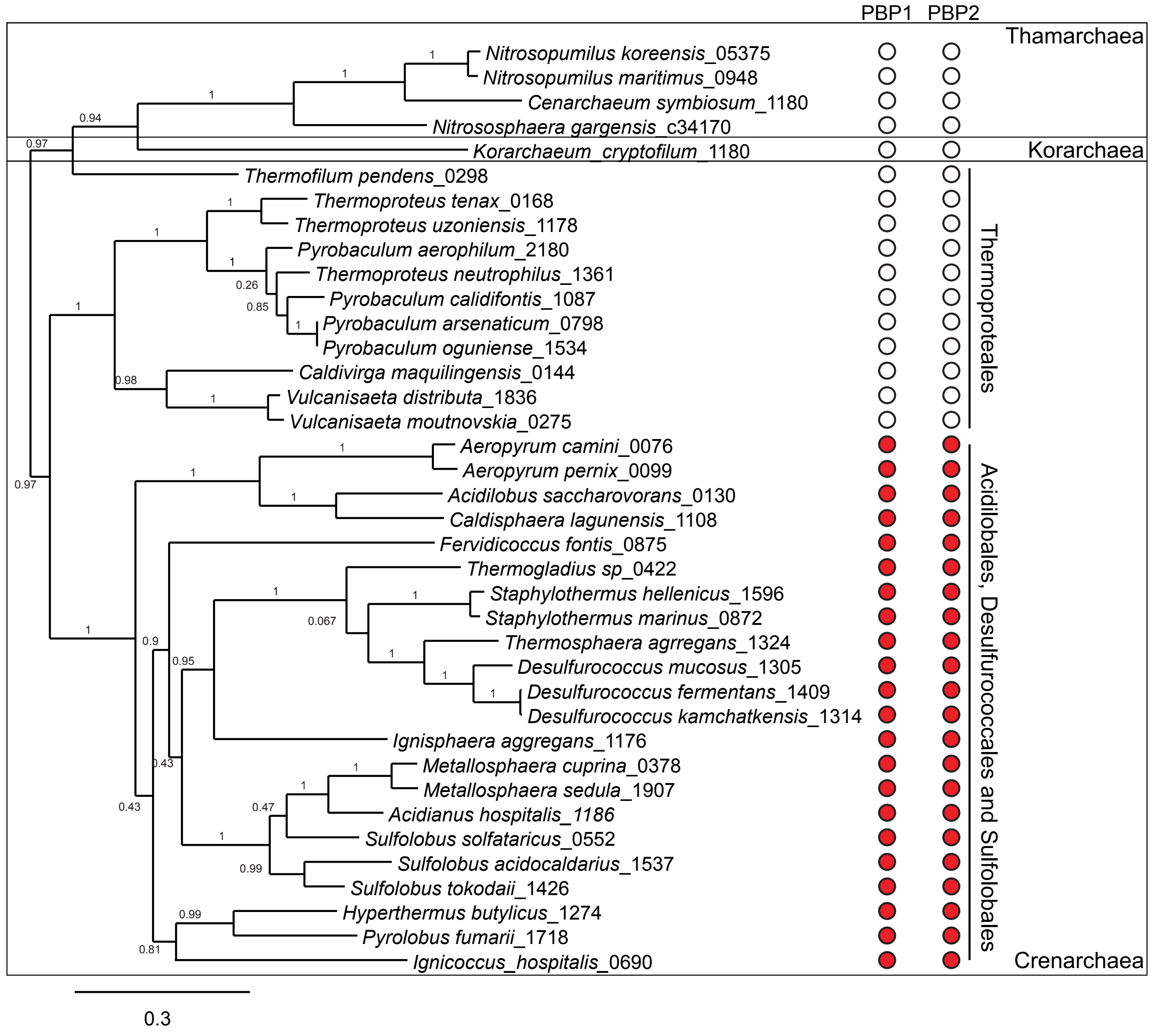

Supplementary Figure 2. Conservation of PBP1 and PBP2.
Phyletic distribution of PBP1 and PBP2 superimposed on a phylogenetic tree of PolB1 orthologs from the indicated archaeal species. The tree was generated using the phyolgeny webserver [1,2]. Red circles indicate the presence of PBP1 or PBP2 orthologs in that species, open circles indicate the lack of detectable homologs.

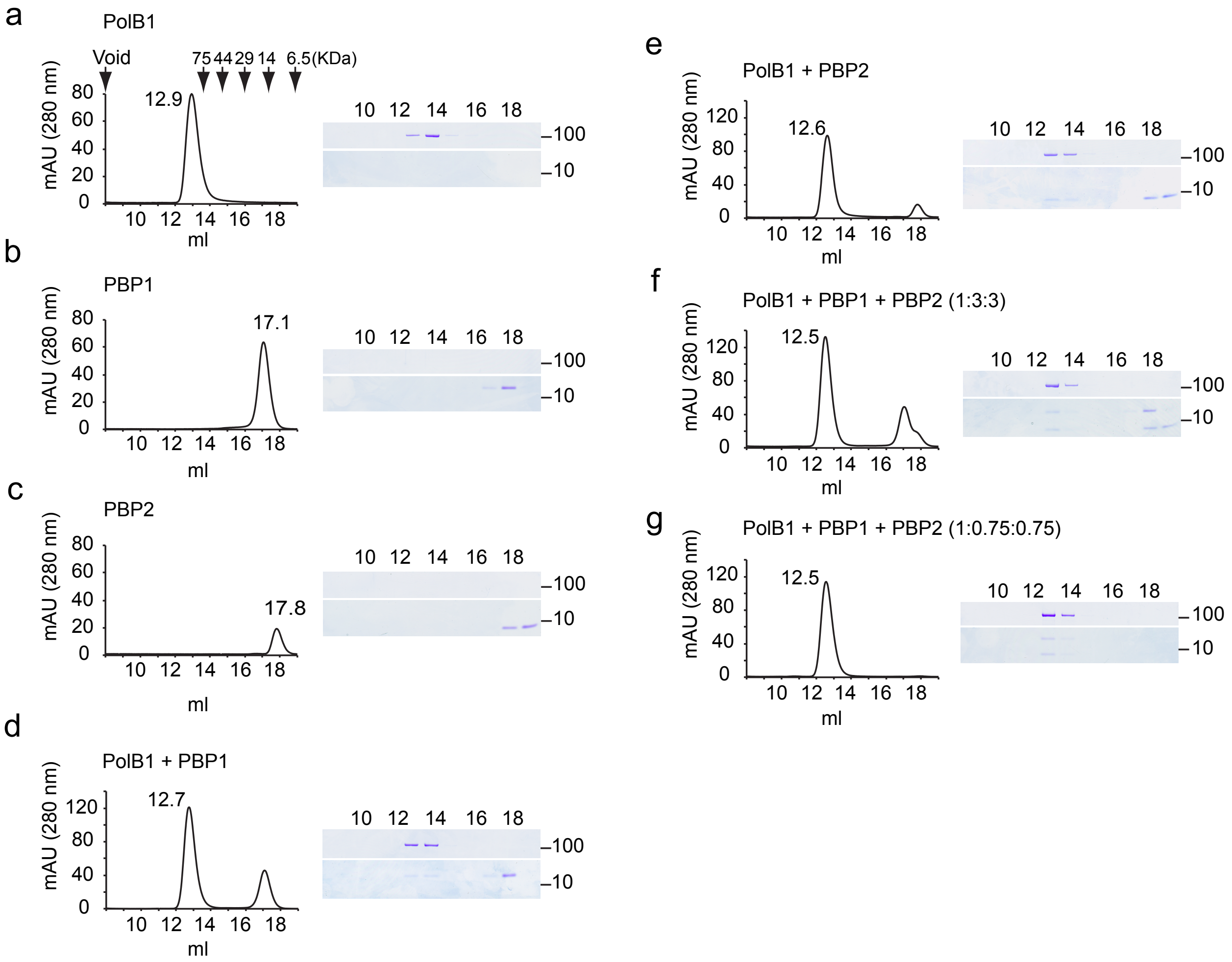

Supplementary Figure 3. Formation of complexes of PolB1 with PBP1 and/or PBP2 . Analytical gel filtration of the indicated proteins and complexes on a Superdex 200 HR 10/300 column. Protein elution was monitored by UV absorbance (280 nm) and the peak elution volume in milliliters is indicated adjacent to the peak. Fractions were also analyzed by gel filtration; size markers are indicated to the right of the panels. Samples were

(a) 0.8 nmol PolB1
(b) 2.4 nmol PBP1
(c) 2.4 nmol PBP2
(d) 0.8 nmol PolB1 + 2.4 nmol PBP1
(e) 0.8 nmol PolB1 + 2.4 nmol PBP2
(f) 0.8 nmol PolB1 + 2.4 nmol PBP1 + 2.4 nmol PBP2
(g) 0.8 nmol PolB1 + 0.6 nmol PBP1 + 0.6 nmol PBP2

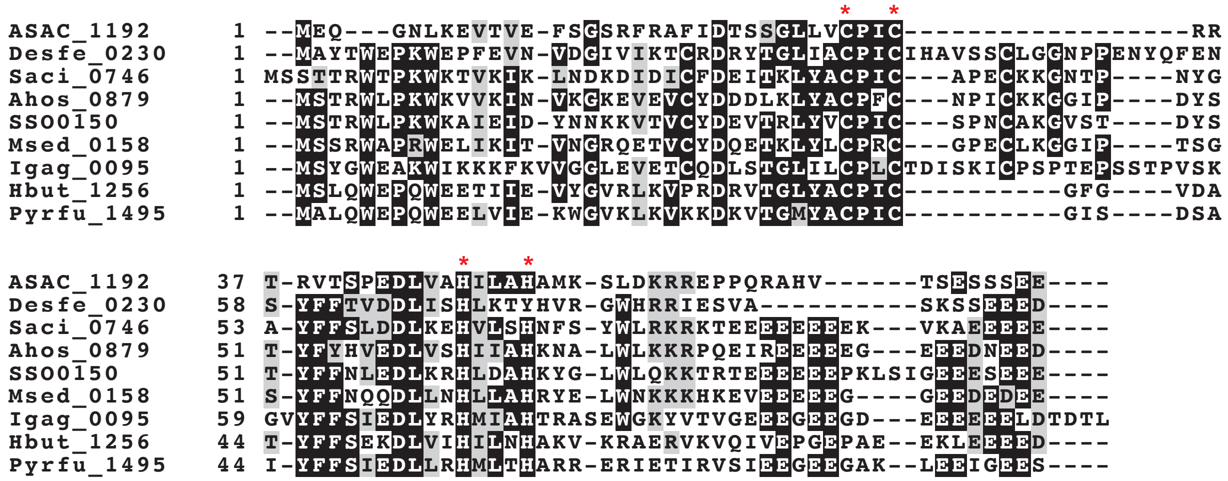

Supplementary Figure 4. Sequence alignment of PBP1 proteins from various archaea. The conserved putative zinc-coordinating residues are indicated by red asterisks. ASAC – *Acidilobus saccharovorans*; Desfe - *Desulfurococcus fermentans*; Saci - *Sulfolobus acidocaldarius*; Ahos – *Acidianus hospitalis*; SSO - *Sulfolobus solfataricus*; Msed - *Metallosphaera sedula*; Igag – *Ignicoccus aggregans*; Hbut – *Hyperthermus butylicus*; Pyrfu- *Pyrolobus fumarii*. The number refers to the open reading frame number in the genome annotations [3].

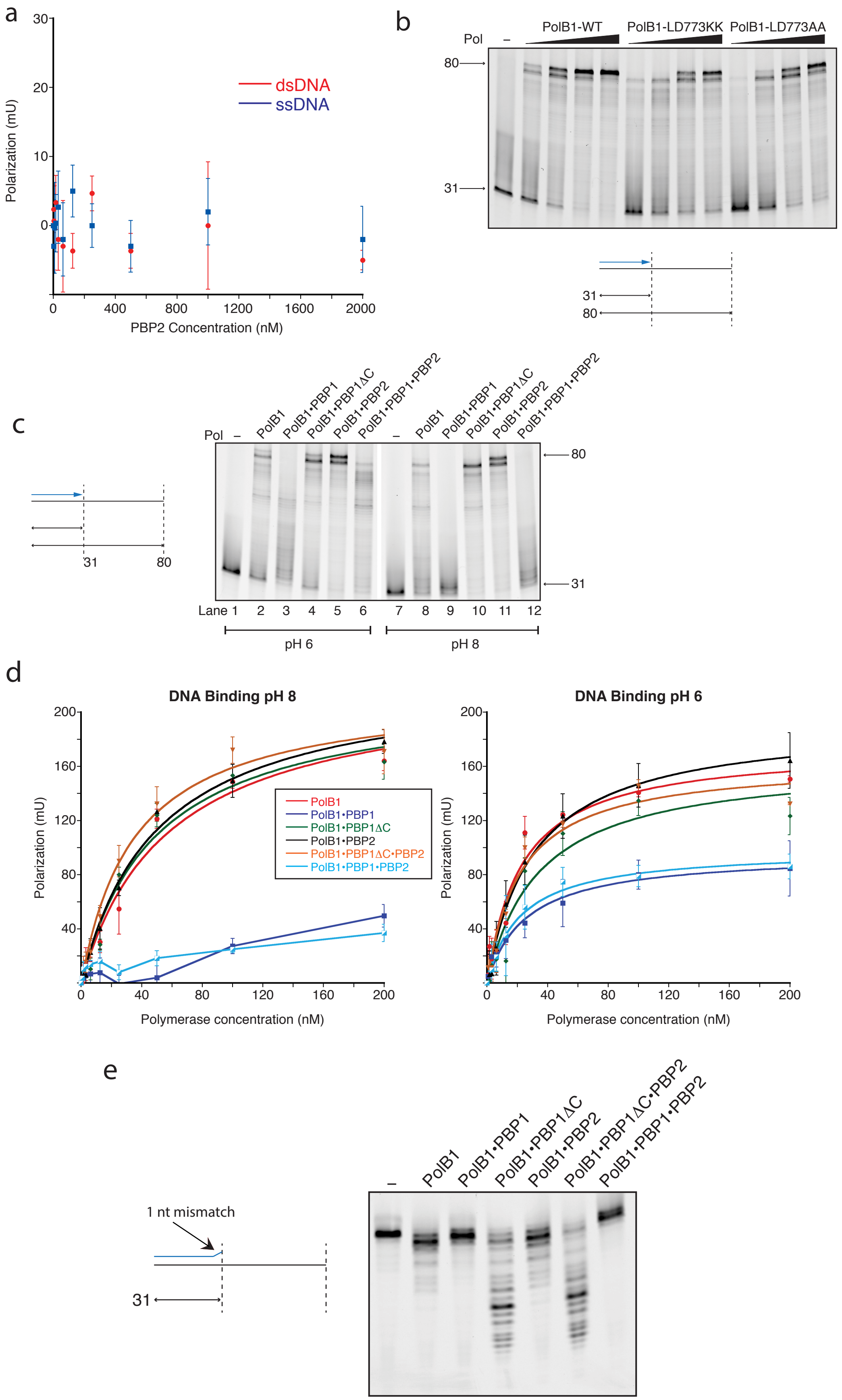
a
dsDNA
ssDNA
Polarization (mU)
PBP2 Concentration (nM)
b
Pol
–
PolB1-WT
PolB1-LD773KK
PolB1-LD773AA
80
31
c
Pol
PolB1
PolB1•PBP1
PolB1•PBP1ΔC
PolB1•PBP2
PolB1•PBP1•PBP2
Lane 1 2 3 4 5 6 7 8 9 10 11 12
pH 6
pH 8
d
DNA Binding pH 8
DNA Binding pH 6
PolB1
PolB1•PBP1
PolB1•PBP1ΔC
PolB1•PBP2
PolB1•PBP1ΔC•PBP2
PolB1•PBP1•PBP2
Polymerase concentration (nM)
e
1 nt mismatch
31

Supplementary Figure 5. Biochemical characterizations of Holoenzyme and sub-complexes.

(a) The ability of PBP2 to bind either single or double stranded DNA was assessed by fluorescence anisotropy measurements. Protein concentrations are a 2-fold dilution series from 2μM. Measurements were performed in triplicate and the error bars are +/-the standard deviation.

(b) Primer extension activity comparison between PolB1 and the thumb domain mutants. Reactions contained 5 nM, 10 nM, 20 nM, 40 nM of each enzyme and 50 nM DNA substrate.

(c) Polymerase activity comparison at pH 6 and 8. Substrate information is illustrated on the left, with the Cy5 labeled primer colored in blue (asterisk indicates the position of labeling). Reactions contained 20 nM enzymes and 50 nM DNA substrate and were incubated at 50 °C.

(d) DNA binding activity comparison at pH 8 and pH 6, determined by fluorescence anisotropy. Polymerase concentrations are 2-fold dilutions from 200 nM to 0.78 nM. Data were plotted using Kaleidagraph version 4.5 and fit to a single site binding model.

(e) Exonuclease activities of PolB1-HE and subassemblies on a DNA substrate with a primer/template containing a mismatch at the 3'-end of the primer. Reactions contained 1 M PolB1-HE and subcomplexes with 1 M DNA substrate. Reaction products were analyzed by denaturing PAGE. 'PBP1ΔC' denotes PBP1 C-terminal tail deletion mutant.

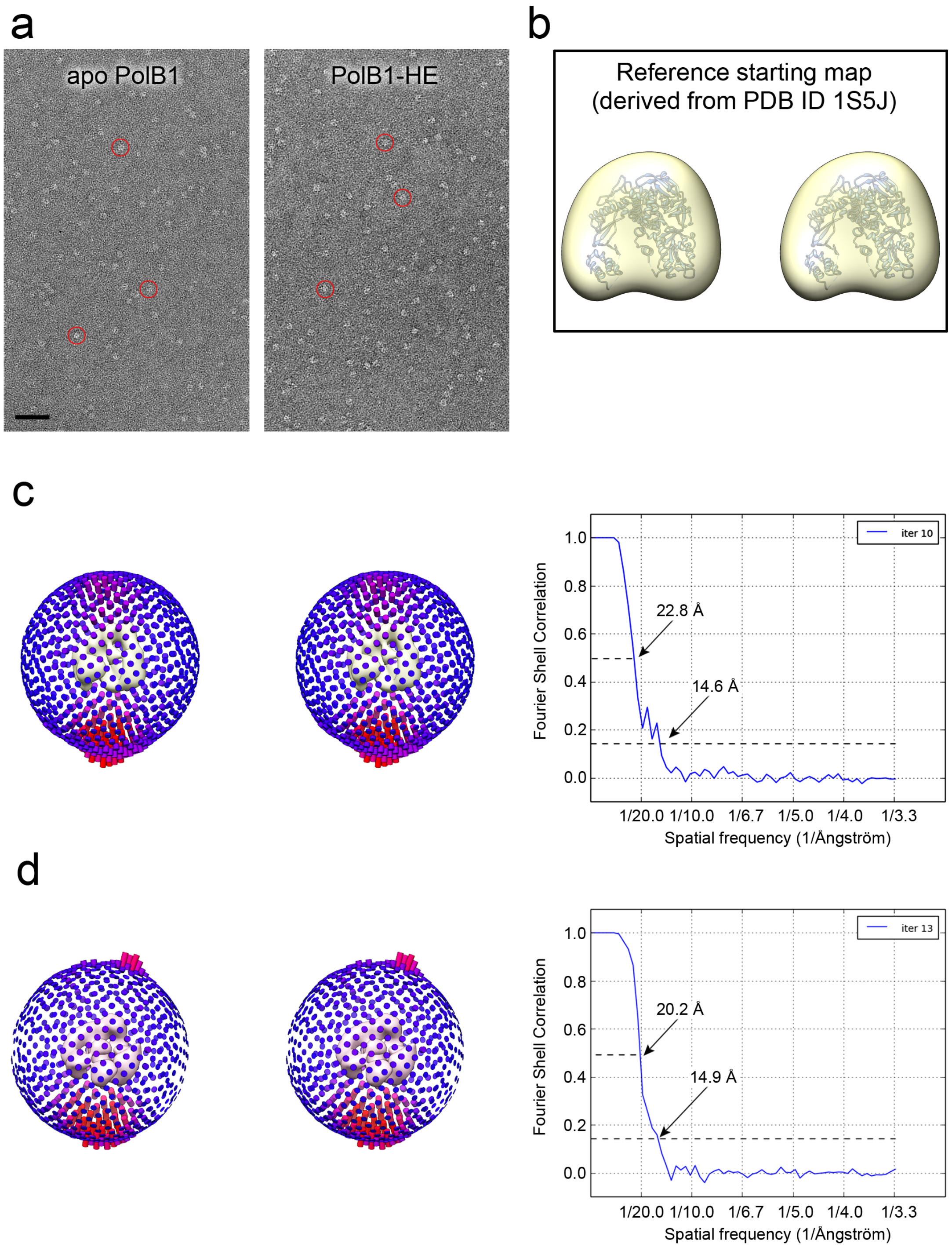

Supplementary Figure 6. Negative stain image processing of apo-PolB1 and PolB1-HE.

(a) Representative area from an electron micrograph recorded for apo-PolB1 (left) and holoenzyme PolB1-HE (right). Red circles mark some of the extracted particles. Scale bar 50 nm.

(b) Stereoview of the volume (semi-transparent yellow) calculated from the crystal structure of DNA PolB1 from *Sulfolobus solfataricus* (PDB ID 1S5J) filtered at 50

Å resolution (contoured at 0.05 threshold in Chimera) with the corresponding atomic model fitted as cartoon representation (colored light-blue).
(c) Left, stereoview of the angular coverage of the particles contributing to the 3D reconstruction for the apo-PolB1 (yellow density in the centre) from Relion and visualized in Chimera; the height of each cylinder relates with the number of particles at that Euler angle. Right, gold-standard Fourier Shell correlation from the last iteration of the auto-3d refinement in Relion and displayed in Scipion with the dashed lines crossing the FSC profile at 0.5 at 22.8 Å resolution and at 0.143 at 14.6 Å resolution respectively.
(d) As (c) but for PolB1-HE holoenzyme (pink density in the center) with FSC calculated at the converged iteration 13. The estimated resolution is 20.2 Å at 0.5 FSC criteria (14.9 Å at 0.143 FSC criteria).

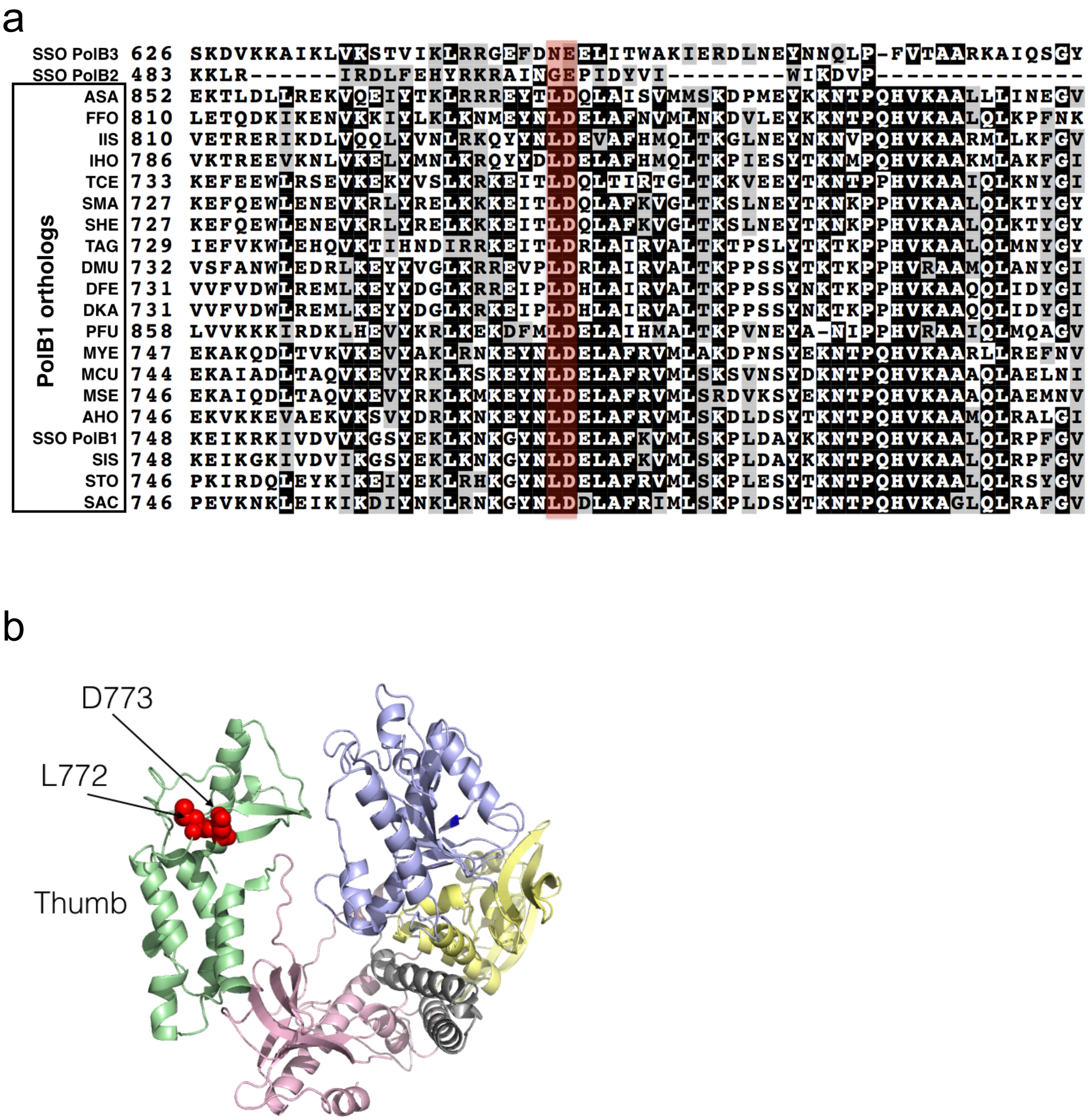

Supplementary Figure 7. Basis of selection of L772 and D773 for mutagenesis

(a) Sequence alignment of protein sequences in the vicinity of the knuckle of the PolB1 thumb domain from a range of members of the Sulfolobales and Desulfurococcales. The leucine and aspartate residues highlighted in red are absolutely conserved in PolB1 but not in PolB2 and PolB3. Accession numbers are given in Supplementary Table 4.

(b) Modeled crystal structure of PolB1 with the leucine and aspartate resides highlighted in part (a) shown in red.

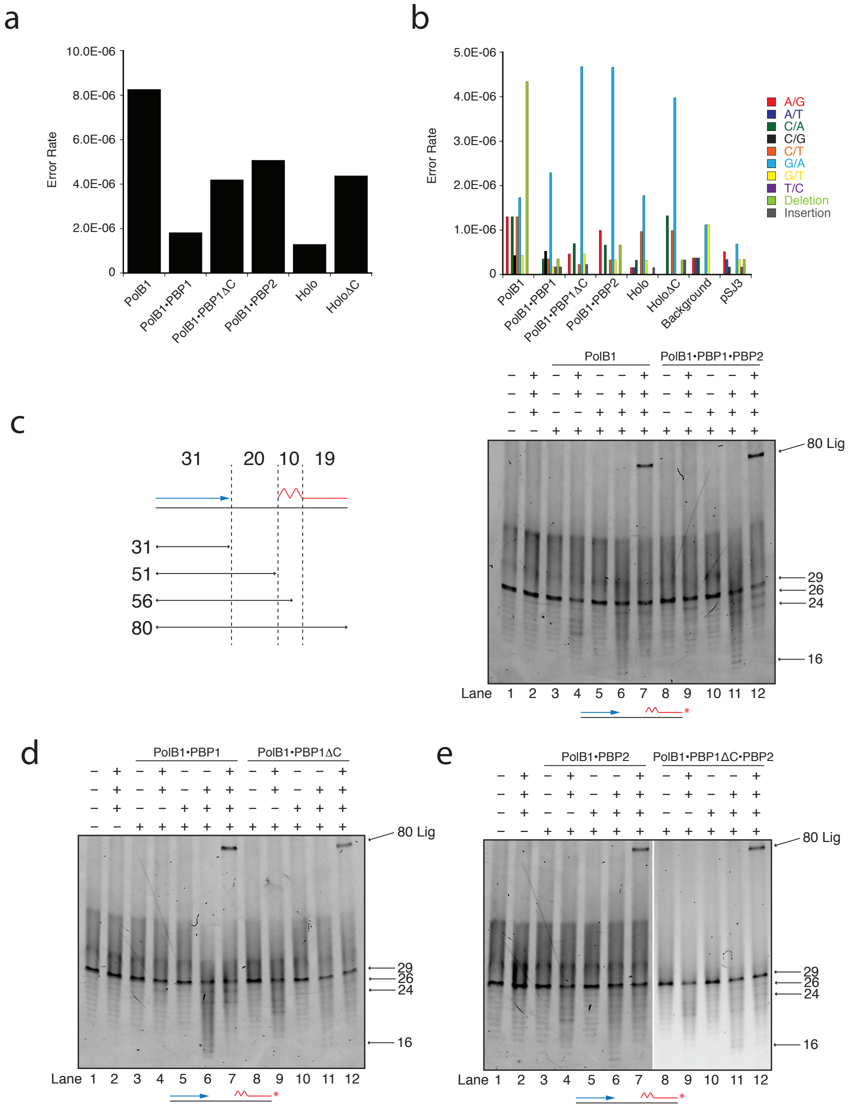

Supplementary Figure 8. DNA polymerase fidelity and lagging strand processing mediated by PolB1 sub-assemblies.

(a) General error rate comparison among PolB1-HE and sub-assemblies. Background mutation rate has been subtracted.

(b) Specific types of error rates generated by all enzymes. Different types of mutations are color-coded. ‘Background’ represents the error rates of gapped pSJ3 plasmid; ‘pSJ3’ represents the error rates of wild type pSJ3.

(c, d and e) The left panel of Figure S8C illustrates the lagging strand synthesis substrate illustration. Primer was labeled at the 5’ end with Cy5 and the downstream “Okazaki fragment” was labeled at the 3’ end with Cy3. Lengths of all the oligos and possible products are labeled. Reactions contained the indicated PolB1 sub-assemblies at 12.5 nM, 200 nM PCNA, 50 nM Fen1, 400 nM Lig1, and 50 nM double-labeled substrate. Reaction products were analyzed by denaturing PAGE. The gels were scanned twice using the Cy3 channel showing Fen1 degradation and Lig1 ligation products. Results for the Cy5 channel are in Figure 7. Notable product sizes are indicated on the right of the gel. The ligation product (80 Lig) is indicated.

	PolB1 N-terminus	PBP1	PBP2
*Nitrososphaera gargensis*_c34170	---MPDRRKRLDDLPELP----	No	No
*Cenarchaeum symbiosum*_1180	--	No	No
*Nitrosopumilus koreensis*_05375	-------------------------------MQVNMGESKKIE----------TMP----	No	No
*Nitrosopumilus maritimus*_0948	-------------------------------MQVNMEETKKIE----------SMP----	No	No
*Korarchaeum cryptofilum*_1180	--MP----	No	No
*Thermofilum pendens*_0298	---MKP----	No	No
*Thermoproteus tenax*_0168	--------------------MAFEEEEFIEEEEVRESEEEEVAEYRIKGALSASIP----	No	No
*Thermoproteus uzoniensis*_1178	-------------------MAFEEEEFLEEGEEIREFEGEEVAEFKIKGAVSTSIP----	No	No
*Pyrobaculum calidifontis*_1087	--------------------MENEFEFEEFEEEVQEYEGEAIEESKLKGVVSVTIP----	No	No
*Thermoproteus neutrophilus*_1361	-------------------MAEFEENFEEFEEEVKEYEGEAIEEGKIKGVVSNSIP----	No	No
*Pyrobaculum aerophilum*_2180	------------------MAEFEEDFEEFEQEEVREYEGEAIEESKIKGVVTSSVP----	No	No
*Pyrobaculum arsenaticum*_0798	--------------------MEFEEEFEEVEEEVTEYEGEAIEEGKIKGVVSNTIP----	No	No
*Pyrobaculum oguniense*_1534	-------------------MVEFEEEFEEVEEEVTEYEGEAIEEGKIKGVVSNTIP----	No	No
*Caldivirga maquilingensis*_0144	-----------------MPKDELDKIEEPEEEEVEESSEEYIQESAITAETPTSTP----	No	No
Vulcanisaeta distributa	-------------MRSKEYREEDEEEEELEEEEFEEREEEEEEEITIKAETPQNTP----	No	No
Vulcanisaeta moutnovskia	-----------------MRSKEDEEEEEIEEEEFEEREEEEEEEITIKAETPQNTP----	No	No
*Aeropyrum camini*_0076	---------MKDSGRKRTTLDESLLSYIRTTVRIKGGAGGGALDDRGRSIDDSEDSEDKT	YES	YES
*Aeropyrum pernix*_0099	-------------MRVRGGQEAAQDKETSLDSDEERGRRGLRQTTLLDYMAGPAKP----	YES	YES
*Acidilobus saccharovorans*_0130	------MALRNDGSPQAGGQGRANEGRSSAASAKSEGKGGGNDRAPAKQPQSQKQP—EP	YES	YES
*Caldisphaera lagunensis*_1108	-------------------------LQEIKVEEMIDKDKNKIINKEDKNYDIINKN----	YES	YES
*Fervidicoccus fontis*_0875	MAMATLKRDRKENGPTLFDFINSSNKKEKTANNFAEDLTYNQDKELKDHEDEVQKL----	YES	YES
*Ignicoccus hospitalis*_0690	----------MKKPRRGPTLLDFLKQKQANDGSKALKAPKPVEEKAPKRPLEGAEK----	YES	YES
*Thermogladius sp*_0422	---------------------------------MKYSKIGDVYTLDFDRYVSTLDS----	YES	YES
*Staphylothermus hellenicus*_1596	------------------------MKYSIIGNVYRLYDVNKVDFIGK-------------	YES	YES
*Staphylothermus marinus*_0872	------------------------MKYNIIGDVYRLYDVNKVDFIDK-------------	YES	YES
*Thermosphaera agrregans*_1324	---------------------------------MRISKIGYVASLPSETYEKVLSK----	YES	YES
*Desulfurococcus mucosus*_1305	--------------------------------MTYMGEQPPPGFIDRSSYKALSKP----	YES	YES
*Desulfurococcus fermentans*_1409	---------------------------------MRSMDILLHVKESFTSYKVLSKP----	YES	YES
*Desulfurococcus kamchatkensis*_13	------------------------------------MDTPLHVKESFTSYKVLSKP----	YES	YES
*Ignicoccus aggregans*_1176	-------------MARQKSIYEFLKKETAKNSDRKPISKDGKEQNNIEIHGSFSND----	YES	YES
*Metallosphaera cuprina*_0378	------------------------MVKQLTLTEFSLPERKMDKIEGKSKEEYIEEP----	YES	YES
*Metallosphaera sedula*_1907	------------------MSIMARQLTLADFSGIKREEPVKQEEKTQEEERPLERP----	YES	YES
*Acidianus hospitalis*_1186	-------------MLKEYFFHLYNVAKQITLFDFSIKQETHSEGKGVEKEEEVQQL----	YES	YES
*Sulfolobus solfataricus*_0552	------------------------MTKQLTLFDIPSSKPAKSEQNTQQSQQSAPVE----	YES	YES
*Sulfolobus acidocaldarius*_1537	------------------------MSKQATLFDFSIKKNESKEQTNQESVEVPKQT----	YES	YES
*Sulfolobus tokodaii*_1426	------------------------MARQITLFDFTLKKEQNKDESRKEEIPHANIN----	YES	YES
*Hyperthermus butylicus*_1274	----------MQRYFDESLRELLTEIRSSRNEANRAGEGARSEKRLSTSVSDNIAD----	YES	YES
*Pyrolobus fumarii*_1718	MARQSTLLEFLNASKRAKRAAGAGGSSPKGTRDTSRKEGGENREKRGEDRNLLEML---L	YES	YES

Supplementary Figure 9. PolB1 in Thermoproteales has an acidic N-terminal tail.

Comparison of the sequences of the N-terminal regions of PolB1 orthologs from the indicated archaeal species. Full PolB1 sequences were aligned using MUSCLE [4], for clarity only the N-terminal regions are shown. Glutamic acid residues are highlighted in red. See also Supplementary Fig. 2 and Fig. 1.

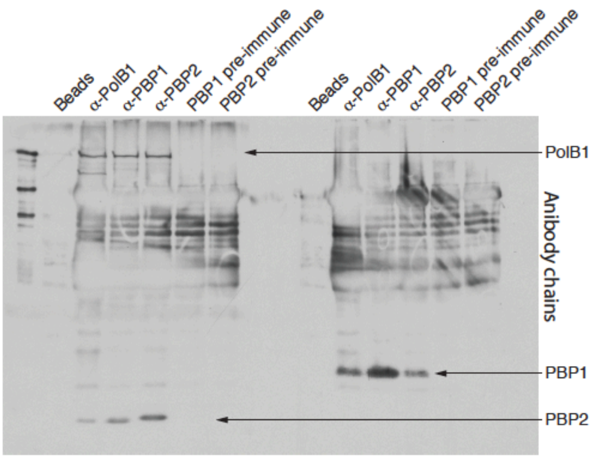

Figure 1b full gel

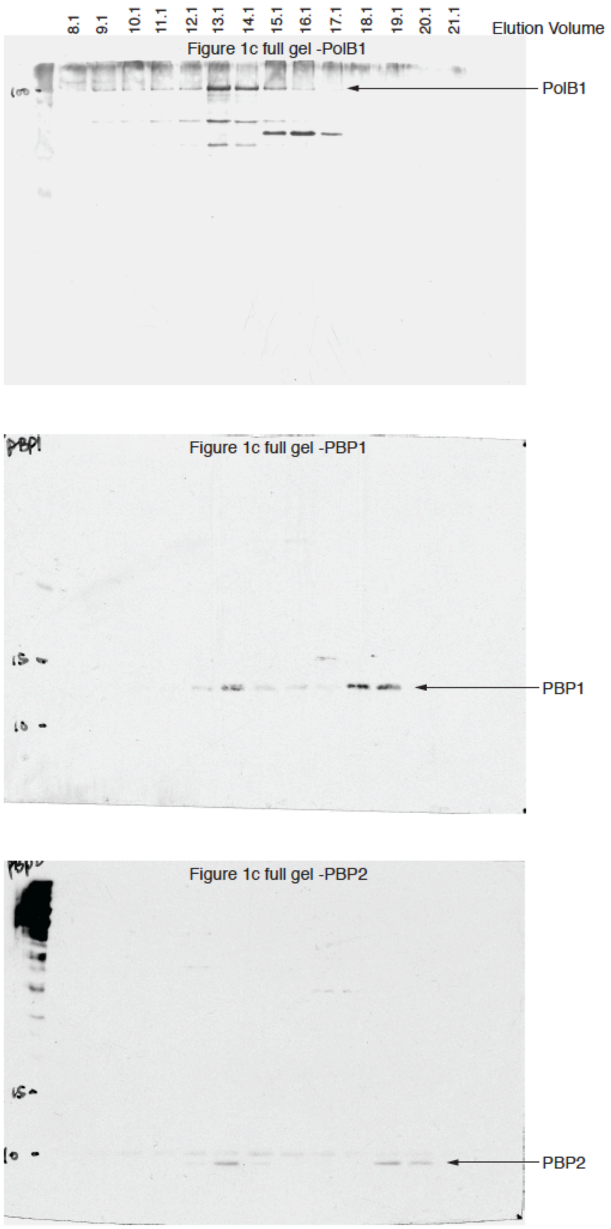

Figure 1c full gel

Supplementary Figure 10. Full membrane images of the cropped western blots presented in Figures 1b and 1c.

Supplementary references

1) Dereeper, A. *et al.,*. Phylogeny.fr: robust phylogenetic analysis for the non-specialist. *Nucleic Acids Res.* **36**, W465-9. (2008)

2) Dereeper, A., Audic, S., Claverie, J.M., Blanc, G. BLAST-EXPLORER helps you building datasets for phylogenetic analysis. *BMC Evol. Biol.* **10**,8. (2010)

3) http://www-archbac.u-psud.fr/projects/sulfolobus/

4) http://www.ebi.ac.uk/Tools/msa/muscle/
